# Supplementary material for: Bivariate genome-wide association analysis strengthens the role of bitter receptor clusters on chromosomes 7 and 12 in human bitter taste
Source: BMC Genomics. 2018 Sep 17;19:678. doi: 10.1186/s12864-018-5058-2 (PMC6142396; doi:10.1186/s12864-018-5058-2)
Supplement: Supplementary file 1 — Table S1. Top 100 SNPs on chromosome 12 associated with the perceived intensity of quinine. (DOCX 177 kb) [file 12864_2018_5058_MOESM1_ESM.docx]

**Table S1. Top 100 SNPs on chromosome 12 associated with the perceived intensity of quinine.**

| **Chr:Position** | **SNP** | **A1/A2** | **MAF** | **Beta** | **SE** | **P** |
| --- | --- | --- | --- | --- | --- | --- |
| 12:11173455 | rs10743937 | C/T | 0.469 | -0.337 | 0.034 | 7.84e-23 |
| 12:11173490 | rs10772419 | C/A | 0.469 | -0.337 | 0.034 | 7.84e-23 |
| 12:11174276 | rs10772420 | G/A | 0.469 | -0.337 | 0.034 | 7.84e-23 |
| 12:11134701 | rs2900554 | T/G | 0.468 | -0.337 | 0.034 | 1.15e-22 |
| 12:11177223 | rs11054173 | A/T | 0.469 | -0.336 | 0.034 | 1.16e-22 |
| 12:11178100 | rs9651854 | C/T | 0.469 | -0.336 | 0.034 | 1.16e-22 |
| 12:11180204 | rs2900578 | T/C | 0.469 | -0.336 | 0.034 | 1.16e-22 |
| 12:11180340 | rs2010481 | A/G | 0.469 | -0.336 | 0.034 | 1.16e-22 |
| 12:11189176 | rs2060705 | G/A | 0.475 | -0.338 | 0.034 | 1.39e-22 |
| 12:11192290 | rs2597981 | G/A | 0.475 | -0.338 | 0.034 | 1.39e-22 |
| 12:11258446 | rs2443739 | C/G | 0.467 | -0.337 | 0.034 | 1.46e-22 |
| 12:11131570 | rs2218820 | T/C | 0.468 | -0.336 | 0.034 | 1.58e-22 |
| 12:11133472 | rs7136588 | T/C | 0.468 | -0.336 | 0.034 | 1.59e-22 |
| 12:11137325 | rs10772396 | T/C | 0.468 | -0.336 | 0.034 | 1.61e-22 |
| 12:11314285 | rs34241192 | C/A | 0.467 | -0.336 | 0.034 | 1.68e-22 |
| 12:11315043 | rs35021653 | T/C | 0.467 | -0.336 | 0.034 | 1.68e-22 |
| 12:11182874 | rs34763234 | G/A | 0.47 | -0.335 | 0.034 | 1.75e-22 |
| 12:11183255 | rs10845293 | G/A | 0.47 | -0.335 | 0.034 | 1.80e-22 |
| 12:11285300 | rs2708371 | C/G | 0.467 | -0.335 | 0.034 | 2.37e-22 |
| 12:11285075 | rs977473 | T/A | 0.467 | -0.335 | 0.034 | 2.38e-22 |
| 12:11285130 | rs1960613 | G/T | 0.467 | -0.335 | 0.034 | 2.38e-22 |
| 12:11308428 | rs61928597 | C/T | 0.467 | -0.335 | 0.034 | 2.42e-22 |
| 12:11308774 | rs35340812 | G/A | 0.467 | -0.335 | 0.034 | 2.42e-22 |
| 12:11309593 | rs35699328 | C/T | 0.467 | -0.335 | 0.034 | 2.42e-22 |
| 12:11310004 | rs61928602 | C/T | 0.467 | -0.335 | 0.034 | 2.44e-22 |
| 12:11310295 | rs3906996 | C/T | 0.467 | -0.335 | 0.034 | 2.44e-22 |
| 12:11311520 | rs7310047 | G/A | 0.467 | -0.335 | 0.034 | 2.44e-22 |
| 12:11312860 | rs6488355 | C/T | 0.467 | -0.335 | 0.034 | 2.44e-22 |
| 12:11312877 | rs6488356 | A/G | 0.467 | -0.335 | 0.034 | 2.44e-22 |
| 12:11312948 | rs7486717 | A/C | 0.467 | -0.335 | 0.034 | 2.44e-22 |
| 12:11313673 | rs7955495 | C/T | 0.467 | -0.335 | 0.034 | 2.47e-22 |
| 12:11266222 | rs2264229 | C/G | 0.466 | -0.335 | 0.034 | 2.47e-22 |
| 12:11253373 | rs2597972 | A/G | 0.467 | -0.335 | 0.034 | 2.49e-22 |
| 12:11307312 | rs34082341 | T/C | 0.467 | -0.334 | 0.034 | 2.54e-22 |
| 12:11307693 | rs7302010 | A/G | 0.467 | -0.334 | 0.034 | 2.54e-22 |
| 12:11306777 | rs7976211 | G/T | 0.467 | -0.334 | 0.034 | 2.54e-22 |
| 12:11307147 | rs35124606 | G/C | 0.467 | -0.334 | 0.034 | 2.58e-22 |
| 12:11306346 | rs34536990 | T/C | 0.467 | -0.334 | 0.034 | 2.60e-22 |
| 12:11263015 | rs2708354 | A/C | 0.467 | -0.335 | 0.034 | 2.74e-22 |
| 12:11293821 | rs35017789 | A/G | 0.468 | -0.333 | 0.034 | 2.83e-22 |
| 12:11294026 | rs7312327 | G/A | 0.468 | -0.333 | 0.034 | 2.83e-22 |
| 12:11294191 | rs7298544 | A/C | 0.468 | -0.333 | 0.034 | 2.83e-22 |
| 12:11296917 | rs61931270 | G/A | 0.468 | -0.333 | 0.034 | 2.83e-22 |
| 12:11296975 | rs36115011 | C/G | 0.468 | -0.333 | 0.034 | 2.83e-22 |
| 12:11297165 | rs34685506 | C/A | 0.468 | -0.333 | 0.034 | 2.83e-22 |
| 12:11297752 | rs6488350 | G/A | 0.468 | -0.333 | 0.034 | 2.83e-22 |
| 12:11297854 | rs6488351 | A/G | 0.468 | -0.333 | 0.034 | 2.83e-22 |
| 12:11299449 | rs35856529 | A/G | 0.468 | -0.333 | 0.034 | 2.83e-22 |
| 12:11299605 | rs35893804 | C/T | 0.468 | -0.333 | 0.034 | 2.83e-22 |
| 12:11299685 | rs34288418 | T/C | 0.468 | -0.333 | 0.034 | 2.83e-22 |
| 12:11299687 | rs34843817 | A/G | 0.468 | -0.333 | 0.034 | 2.83e-22 |
| 12:11299851 | rs34927715 | A/G | 0.468 | -0.333 | 0.034 | 2.83e-22 |
| 12:11299977 | rs61931278 | G/C | 0.468 | -0.333 | 0.034 | 2.83e-22 |
| 12:11300006 | rs61931279 | C/T | 0.468 | -0.333 | 0.034 | 2.83e-22 |
| 12:11300369 | rs7973730 | C/T | 0.468 | -0.333 | 0.034 | 2.83e-22 |
| 12:11304086 | rs61928564 | T/A | 0.468 | -0.333 | 0.034 | 2.83e-22 |
| 12:11304159 | rs61928565 | G/T | 0.468 | -0.333 | 0.034 | 2.83e-22 |
| 12:11304327 | rs61928566 | T/C | 0.468 | -0.333 | 0.034 | 2.83e-22 |
| 12:11305373 | rs34548551 | G/C | 0.468 | -0.333 | 0.034 | 2.83e-22 |
| 12:11305514 | rs34769150 | G/T | 0.468 | -0.333 | 0.034 | 2.83e-22 |
| 12:11296329 | rs35280352 | C/T | 0.468 | -0.333 | 0.034 | 2.84e-22 |
| 12:11305724 | rs7959320 | A/G | 0.468 | -0.333 | 0.034 | 2.88e-22 |
| 12:11306064 | rs7965506 | T/C | 0.468 | -0.333 | 0.034 | 2.88e-22 |
| 12:11198678 | rs2708322 | T/C | 0.468 | -0.334 | 0.034 | 2.91e-22 |
| 12:11204944 | rs2597992 | T/A | 0.468 | -0.333 | 0.034 | 2.96e-22 |
| 12:11206217 | rs2597994 | A/G | 0.468 | -0.333 | 0.034 | 2.96e-22 |
| 12:11207864 | rs2708386 | A/C | 0.468 | -0.333 | 0.034 | 2.96e-22 |
| 12:11208989 | rs2597998 | C/T | 0.468 | -0.333 | 0.034 | 2.96e-22 |
| 12:11209938 | rs2598000 | T/C | 0.468 | -0.333 | 0.034 | 2.96e-22 |
| 12:11211904 | rs2708383 | C/T | 0.468 | -0.333 | 0.034 | 2.96e-22 |
| 12:11250938 | rs2443094 | A/G | 0.468 | -0.333 | 0.034 | 2.96e-22 |
| 12:11256413 | rs2597966 | C/T | 0.467 | -0.334 | 0.034 | 3.01e-22 |
| 12:11256437 | rs2600332 | T/C | 0.467 | -0.334 | 0.034 | 3.01e-22 |
| 12:11256660 | rs2708361 | C/T | 0.467 | -0.334 | 0.034 | 3.01e-22 |
| 12:11257001 | rs2597965 | A/G | 0.467 | -0.334 | 0.034 | 3.01e-22 |
| 12:11257149 | rs2600341 | T/C | 0.467 | -0.334 | 0.034 | 3.01e-22 |
| 12:11257235 | rs2597964 | A/G | 0.467 | -0.334 | 0.034 | 3.01e-22 |
| 12:11257876 | rs2708359 | T/A | 0.467 | -0.334 | 0.034 | 3.01e-22 |
| 12:11258011 | rs2597963 | C/T | 0.467 | -0.334 | 0.034 | 3.01e-22 |
| 12:11258149 | rs2600349 | C/T | 0.467 | -0.334 | 0.034 | 3.01e-22 |
| 12:11258253 | rs2708358 | C/T | 0.467 | -0.334 | 0.034 | 3.01e-22 |
| 12:11259871 | rs2599405 | C/G | 0.467 | -0.334 | 0.034 | 3.01e-22 |
| 12:11259894 | rs2597960 | A/G | 0.467 | -0.334 | 0.034 | 3.01e-22 |
| 12:11260410 | rs2597959 | A/C | 0.467 | -0.334 | 0.034 | 3.01e-22 |
| 12:11260644 | rs2600338 | G/A | 0.467 | -0.334 | 0.034 | 3.01e-22 |
| 12:11260761 | rs2600339 | G/C | 0.467 | -0.334 | 0.034 | 3.01e-22 |
| 12:11261123 | rs2708356 | G/A | 0.467 | -0.334 | 0.034 | 3.01e-22 |
| 12:11261967 | rs2600342 | T/G | 0.467 | -0.334 | 0.034 | 3.01e-22 |
| 12:11262086 | rs2597952 | T/C | 0.467 | -0.334 | 0.034 | 3.01e-22 |
| 12:11262265 | rs2599411 | A/G | 0.467 | -0.334 | 0.034 | 3.01e-22 |
| 12:11262724 | rs2597951 | G/C | 0.467 | -0.334 | 0.034 | 3.01e-22 |
| 12:11262955 | rs2597950 | T/C | 0.467 | -0.334 | 0.034 | 3.01e-22 |
| 12:11263089 | rs2597949 | G/A | 0.467 | -0.334 | 0.034 | 3.01e-22 |
| 12:11263112 | rs2708353 | A/G | 0.467 | -0.334 | 0.034 | 3.01e-22 |
| 12:11263318 | rs2597947 | T/C | 0.467 | -0.334 | 0.034 | 3.01e-22 |
| 12:11263744 | rs75814885 | T/G | 0.467 | -0.334 | 0.034 | 3.01e-22 |
| 12:11264798 | rs2708352 | T/C | 0.467 | -0.334 | 0.034 | 3.01e-22 |
| 12:11264980 | rs2264190 | G/A | 0.467 | -0.334 | 0.034 | 3.01e-22 |
| 12:11265851 | rs2600344 | C/T | 0.467 | -0.334 | 0.034 | 3.01e-22 |
| 12:11266342 | rs2264192 | C/T | 0.467 | -0.334 | 0.034 | 3.01e-22 |
