# Supplementary material for: Bivariate genome-wide association analysis strengthens the role of bitter receptor clusters on chromosomes 7 and 12 in human bitter taste
Source: BMC Genomics. 2018 Sep 17;19:678. doi: 10.1186/s12864-018-5058-2 (PMC6142396; doi:10.1186/s12864-018-5058-2)
Supplement: Supplementary file 2 — Table S2. Top 100 SNPs on chromosome 12 associated with the perceived intensity of caffeine. (DOCX 178 kb) [file 12864_2018_5058_MOESM2_ESM.docx]

**Table S2. Top 100 SNPs on chromosome 12 associated with the perceived intensity of caffeine.**

| **Chr:Position** | **SNP** | **A1/A2** | **MAF** | **Beta** | **SE** | **P** |
| --- | --- | --- | --- | --- | --- | --- |
| 12:11189966 | rs2597979 | G/C | 0.163 | 0.26 | 0.05 | 4.17e-08 |
| 12:11351229 | rs1669436 | C/G | 0.139 | 0.28 | 0.05 | 8.98e-08 |
| 12:11174753 | rs1868769 | G/A | 0.194 | 0.23 | 0.04 | 9.18e-08 |
| 12:11180299 | rs2084648 | A/T | 0.193 | 0.23 | 0.04 | 1.04e-07 |
| 12:11183451 | rs10743938 | A/T | 0.193 | 0.23 | 0.04 | 1.09e-07 |
| 12:11184140 | rs10845296 | G/A | 0.193 | 0.23 | 0.04 | 1.09e-07 |
| 12:11203065 | rs2597988 | T/C | 0.145 | 0.26 | 0.05 | 1.12e-07 |
| 12:11329548 | rs73053413 | T/C | 0.14 | 0.27 | 0.05 | 1.21e-07 |
| 12:11139589 | rs10772398 | C/T | 0.147 | 0.26 | 0.05 | 1.24e-07 |
| 12:11128666 | rs10772395 | C/T | 0.147 | 0.26 | 0.05 | 1.28e-07 |
| 12:11131462 | rs6488333 | C/T | 0.147 | 0.26 | 0.05 | 1.28e-07 |
| 12:11202522 | rs2257110 | G/C | 0.145 | 0.26 | 0.05 | 1.30e-07 |
| 12:11293130 | rs2600337 | G/C | 0.14 | 0.27 | 0.05 | 1.37e-07 |
| 12:11198433 | rs2708323 | A/G | 0.145 | 0.26 | 0.05 | 1.64e-07 |
| 12:11199734 | rs2597984 | T/C | 0.145 | 0.26 | 0.05 | 1.64e-07 |
| 12:11345936 | rs1650024 | A/G | 0.141 | 0.27 | 0.05 | 1.91e-07 |
| 12:11345005 | rs1669421 | T/C | 0.141 | 0.26 | 0.05 | 2.21e-07 |
| 12:11345136 | rs1650022 | T/G | 0.141 | 0.26 | 0.05 | 2.21e-07 |
| 12:11346562 | rs1669424 | T/C | 0.141 | 0.26 | 0.05 | 2.21e-07 |
| 12:11083677 | rs10772391 | T/C | 0.144 | 0.26 | 0.05 | 2.29e-07 |
| 12:11347219 | rs1669425 | A/G | 0.141 | 0.26 | 0.05 | 2.33e-07 |
| 12:11347223 | rs1650025 | G/C | 0.141 | 0.26 | 0.05 | 2.33e-07 |
| 12:11309537 | rs34692077 | T/C | 0.143 | 0.26 | 0.05 | 2.51e-07 |
| 12:11347649 | rs1669426 | T/C | 0.141 | 0.26 | 0.05 | 2.53e-07 |
| 12:11347716 | rs1650026 | T/C | 0.141 | 0.26 | 0.05 | 2.53e-07 |
| 12:11347751 | rs1650027 | A/G | 0.141 | 0.26 | 0.05 | 2.53e-07 |
| 12:11347798 | rs1650028 | A/G | 0.141 | 0.26 | 0.05 | 2.53e-07 |
| 12:11348296 | rs1427754 | C/T | 0.141 | 0.26 | 0.05 | 2.53e-07 |
| 12:11348862 | rs1669430 | T/C | 0.141 | 0.26 | 0.05 | 2.53e-07 |
| 12:11348886 | rs1669431 | G/T | 0.141 | 0.26 | 0.05 | 2.53e-07 |
| 12:11349622 | rs1650032 | A/G | 0.141 | 0.26 | 0.05 | 2.53e-07 |
| 12:11349671 | rs2600373 | A/G | 0.141 | 0.26 | 0.05 | 2.53e-07 |
| 12:11349938 | rs1669434 | T/G | 0.141 | 0.26 | 0.05 | 2.53e-07 |
| 12:11350263 | rs1669435 | T/C | 0.141 | 0.26 | 0.05 | 2.53e-07 |
| 12:11350661 | rs1650033 | T/C | 0.141 | 0.26 | 0.05 | 2.53e-07 |
| 12:11350951 | rs1650034 | T/G | 0.141 | 0.26 | 0.05 | 2.53e-07 |
| 12:11350963 | rs1650035 | T/G | 0.141 | 0.26 | 0.05 | 2.53e-07 |
| 12:11345572 | rs1650023 | A/G | 0.141 | 0.26 | 0.05 | 2.54e-07 |
| 12:11342401 | rs1669415 | C/T | 0.141 | 0.26 | 0.05 | 2.63e-07 |
| 12:11342415 | rs1669416 | G/C | 0.141 | 0.26 | 0.05 | 2.63e-07 |
| 12:11342525 | rs1669417 | G/A | 0.141 | 0.26 | 0.05 | 2.63e-07 |
| 12:11343422 | rs1669419 | G/A | 0.141 | 0.26 | 0.05 | 2.63e-07 |
| 12:11349605 | rs1669432 | G/T | 0.14 | 0.26 | 0.05 | 2.64e-07 |
| 12:11349732 | rs1669433 | A/G | 0.14 | 0.26 | 0.05 | 2.64e-07 |
| 12:11311787 | rs61928603 | C/T | 0.148 | 0.26 | 0.05 | 2.70e-07 |
| 12:11343964 | rs1650021 | T/A | 0.141 | 0.26 | 0.05 | 2.79e-07 |
| 12:11203459 | rs2597990 | G/A | 0.144 | 0.26 | 0.05 | 2.86e-07 |
| 12:11098139 | rs2418223 | T/A | 0.147 | 0.25 | 0.05 | 3.05e-07 |
| 12:11341878 | rs61928650 | T/C | 0.141 | 0.26 | 0.05 | 3.16e-07 |
| 12:11215852 | rs1817043 | G/A | 0.144 | 0.26 | 0.05 | 3.31e-07 |
| 12:11216315 | rs2708377 | C/T | 0.144 | 0.26 | 0.05 | 3.31e-07 |
| 12:11216972 | rs2255418 | C/T | 0.144 | 0.26 | 0.05 | 3.31e-07 |
| 12:11217237 | rs2599415 | G/A | 0.144 | 0.26 | 0.05 | 3.31e-07 |
| 12:11338781 | rs1669413 | C/A | 0.142 | 0.26 | 0.05 | 3.59e-07 |
| 12:11079998 | rs10743936 | C/T | 0.147 | 0.25 | 0.05 | 3.68e-07 |
| 12:11307615 | rs4763634 | T/C | 0.142 | 0.26 | 0.05 | 3.88e-07 |
| 12:11164751 | rs7315843 | G/A | 0.148 | 0.25 | 0.05 | 3.93e-07 |
| 12:11165233 | rs1376249 | A/T | 0.148 | 0.25 | 0.05 | 3.93e-07 |
| 12:11324559 | rs8181 | C/G | 0.141 | 0.26 | 0.05 | 4.07e-07 |
| 12:11326071 | rs2900127 | G/A | 0.141 | 0.26 | 0.05 | 4.07e-07 |
| 12:11326315 | rs4763637 | T/C | 0.141 | 0.26 | 0.05 | 4.07e-07 |
| 12:11328768 | rs1650051 | G/A | 0.141 | 0.26 | 0.05 | 4.07e-07 |
| 12:11329053 | rs1669406 | G/A | 0.141 | 0.26 | 0.05 | 4.07e-07 |
| 12:11329249 | rs1669407 | C/T | 0.141 | 0.26 | 0.05 | 4.07e-07 |
| 12:11331479 | rs187328 | T/C | 0.141 | 0.26 | 0.05 | 4.07e-07 |
| 12:11331726 | rs319266 | G/A | 0.141 | 0.26 | 0.05 | 4.07e-07 |
| 12:11332584 | rs319269 | C/A | 0.142 | 0.26 | 0.05 | 4.09e-07 |
| 12:11333542 | rs319270 | C/A | 0.142 | 0.26 | 0.05 | 4.09e-07 |
| 12:11337442 | rs319277 | G/A | 0.142 | 0.26 | 0.05 | 4.09e-07 |
| 12:11338983 | rs1650019 | T/C | 0.141 | 0.26 | 0.05 | 4.15e-07 |
| 12:11316437 | rs61928609 | A/C | 0.142 | 0.26 | 0.05 | 4.18e-07 |
| 12:11165540 | rs7306087 | A/C | 0.149 | 0.25 | 0.05 | 4.19e-07 |
| 12:11167674 | rs2900577 | G/C | 0.149 | 0.25 | 0.05 | 4.19e-07 |
| 12:11307278 | rs4763632 | A/G | 0.142 | 0.26 | 0.05 | 4.24e-07 |
| 12:11315112 | rs61928606 | G/A | 0.141 | 0.26 | 0.05 | 4.29e-07 |
| 12:11150579 | rs1463237 | C/T | 0.148 | 0.25 | 0.05 | 4.34e-07 |
| 12:11305844 | rs7962445 | T/C | 0.142 | 0.26 | 0.05 | 4.38e-07 |
| 12:11119119 | rs6488331 | T/C | 0.159 | 0.24 | 0.05 | 4.39e-07 |
| 12:11320130 | rs4763636 | A/G | 0.142 | 0.26 | 0.05 | 4.40e-07 |
| 12:11320643 | rs61928615 | A/G | 0.142 | 0.26 | 0.05 | 4.40e-07 |
| 12:11324401 | rs1047713 | C/G | 0.142 | 0.26 | 0.05 | 4.53e-07 |
| 12:11320297 | rs61928614 | A/G | 0.142 | 0.26 | 0.05 | 4.58e-07 |
| 12:11311159 | rs7298947 | T/C | 0.142 | 0.26 | 0.05 | 4.59e-07 |
| 12:11311590 | rs7296270 | A/T | 0.142 | 0.26 | 0.05 | 4.59e-07 |
| 12:11311947 | rs61928604 | C/T | 0.142 | 0.26 | 0.05 | 4.59e-07 |
| 12:11313886 | rs7973298 | T/C | 0.142 | 0.26 | 0.05 | 4.59e-07 |
| 12:11150551 | rs4388985 | G/A | 0.149 | 0.25 | 0.05 | 4.61e-07 |
| 12:11158728 | rs10772414 | C/G | 0.149 | 0.25 | 0.05 | 4.61e-07 |
| 12:11171846 | rs10734843 | A/G | 0.149 | 0.25 | 0.05 | 4.61e-07 |
| 12:11309750 | rs35318883 | T/C | 0.141 | 0.26 | 0.05 | 4.62e-07 |
| 12:11091432 | rs3741843 | C/T | 0.147 | 0.25 | 0.05 | 4.72e-07 |
| 12:11252797 | rs2597975 | C/T | 0.143 | 0.25 | 0.05 | 5.06e-07 |
| 12:11252845 | rs2597974 | T/C | 0.143 | 0.25 | 0.05 | 5.06e-07 |
| 12:11338555 | rs1669409 | A/T | 0.142 | 0.26 | 0.05 | 5.06e-07 |
| 12:11267880 | rs2600347 | G/A | 0.142 | 0.25 | 0.05 | 5.41e-07 |
| 12:11299218 | rs7487324 | C/T | 0.142 | 0.25 | 0.05 | 5.42e-07 |
| 12:11300255 | rs61931280 | C/T | 0.142 | 0.25 | 0.05 | 5.42e-07 |
| 12:11304413 | rs61928567 | T/C | 0.142 | 0.25 | 0.05 | 5.42e-07 |
| 12:11170837 | rs10732561 | T/G | 0.148 | 0.24 | 0.05 | 5.68e-07 |
| 12:11261589 | rs2600340 | T/C | 0.143 | 0.25 | 0.05 | 5.71e-07 |
