# Supplementary material for: Bivariate genome-wide association analysis strengthens the role of bitter receptor clusters on chromosomes 7 and 12 in human bitter taste
Source: BMC Genomics. 2018 Sep 17;19:678. doi: 10.1186/s12864-018-5058-2 (PMC6142396; doi:10.1186/s12864-018-5058-2)
Supplement: Supplementary file 3 — Table S3. Top 100 SNPs on chromosome 12 associated with the perceived intensity of sucrose octaacetate (SOA). (DOCX 181 kb) [file 12864_2018_5058_MOESM3_ESM.docx]

**Table S3. Top 100 SNPs on chromosome 12 associated with the perceived intensity of sucrose octaacetate (SOA).**

| **Chr:Position** | **SNP** | **A1/A2** | **MAF** | **Beta** | **SE** | **P** |
| --- | --- | --- | --- | --- | --- | --- |
| 12:11194384 | rs67487380 | A/G | 0.275 | -0.202 | 0.040 | 3.78e-07 |
| 12:11195162 | rs1901188 | T/C | 0.275 | -0.202 | 0.040 | 3.78e-07 |
| 12:11170152 | rs10845291 | T/C | 0.274 | -0.197 | 0.040 | 7.52e-07 |
| 12:11196583 | rs7310224 | G/A | 0.264 | -0.198 | 0.040 | 1.09e-06 |
| 12:11195322 | rs35969800 | A/G | 0.265 | -0.197 | 0.040 | 1.14e-06 |
| 12:11211734 | rs35846189 | C/A | 0.321 | -0.183 | 0.038 | 1.66e-06 |
| 12:11211781 | rs35413384 | C/G | 0.321 | -0.183 | 0.038 | 1.66e-06 |
| 12:11220455 | rs11526041 | A/C | 0.321 | -0.183 | 0.038 | 1.75e-06 |
| 12:11205292 | rs2900581 | G/A | 0.321 | -0.182 | 0.038 | 1.89e-06 |
| 12:11205343 | rs2900582 | A/G | 0.321 | -0.182 | 0.038 | 1.89e-06 |
| 12:11166968 | rs7310849 | G/A | 0.32 | -0.181 | 0.038 | 1.99e-06 |
| 12:11272192 | rs7313683 | G/T | 0.348 | -0.185 | 0.039 | 2.06e-06 |
| 12:11252729 | rs35097305 | C/A | 0.32 | -0.182 | 0.038 | 2.16e-06 |
| 12:11315644 | rs1349553 | G/A | 0.315 | -0.185 | 0.039 | 2.23e-06 |
| 12:11167763 | rs10845290 | A/G | 0.284 | -0.187 | 0.039 | 2.32e-06 |
| 12:11175414 | rs4763235 | G/C | 0.321 | -0.179 | 0.038 | 2.52e-06 |
| 12:11256031 | rs28419178 | T/C | 0.319 | -0.181 | 0.038 | 2.65e-06 |
| 12:11138852 | rs1376251 | T/C | 0.321 | -0.179 | 0.038 | 2.80e-06 |
| 12:11141752 | rs2418302 | T/C | 0.321 | -0.179 | 0.038 | 2.80e-06 |
| 12:11314022 | rs35746980 | T/C | 0.315 | -0.183 | 0.039 | 2.82e-06 |
| 12:11318574 | rs1551193 | A/C | 0.314 | -0.183 | 0.039 | 3.00e-06 |
| 12:11147660 | rs11054140 | T/C | 0.321 | -0.178 | 0.038 | 3.05e-06 |
| 12:11259611 | rs66840927 | T/G | 0.319 | -0.180 | 0.039 | 3.26e-06 |
| 12:11166536 | rs11054164 | T/C | 0.32 | -0.177 | 0.038 | 3.47e-06 |
| 12:11166578 | rs1901190 | C/T | 0.32 | -0.177 | 0.038 | 3.47e-06 |
| 12:11143223 | rs12296784 | A/C | 0.266 | -0.186 | 0.040 | 3.83e-06 |
| 12:11272738 | rs3851590 | C/G | 0.261 | -0.194 | 0.042 | 3.95e-06 |
| 12:11131212 | rs2900553 | T/G | 0.32 | -0.176 | 0.038 | 4.11e-06 |
| 12:11131791 | rs7138953 | A/G | 0.32 | -0.176 | 0.038 | 4.11e-06 |
| 12:11177580 | rs10772421 | A/C | 0.266 | -0.185 | 0.040 | 4.25e-06 |
| 12:11262180 | rs4763627 | A/G | 0.318 | -0.178 | 0.039 | 4.38e-06 |
| 12:11311958 | rs2290318 | C/G | 0.315 | -0.179 | 0.039 | 4.42e-06 |
| 12:11312026 | rs2290319 | A/C | 0.315 | -0.179 | 0.039 | 4.43e-06 |
| 12:11263238 | rs4763628 | C/A | 0.319 | -0.178 | 0.039 | 4.59e-06 |
| 12:11304132 | rs34373518 | G/A | 0.317 | -0.178 | 0.039 | 4.60e-06 |
| 12:11263799 | rs112665659 | C/A | 0.319 | -0.178 | 0.039 | 4.61e-06 |
| 12:11263373 | rs4763629 | G/A | 0.319 | -0.178 | 0.039 | 4.68e-06 |
| 12:11309606 | rs34274000 | A/G | 0.315 | -0.179 | 0.039 | 4.78e-06 |
| 12:11291030 | rs7316032 | G/A | 0.317 | -0.177 | 0.039 | 5.32e-06 |
| 12:11293408 | rs34708147 | G/A | 0.317 | -0.177 | 0.039 | 5.32e-06 |
| 12:11299571 | rs34666803 | C/T | 0.317 | -0.177 | 0.039 | 5.32e-06 |
| 12:11281517 | rs67961444 | C/A | 0.317 | -0.177 | 0.039 | 5.46e-06 |
| 12:11285233 | rs7980677 | C/T | 0.317 | -0.177 | 0.039 | 5.48e-06 |
| 12:11289324 | rs6488346 | C/T | 0.317 | -0.177 | 0.039 | 5.48e-06 |
| 12:11264628 | rs35376087 | T/A | 0.318 | -0.175 | 0.039 | 6.12e-06 |
| 12:11266214 | rs145696441 | C/G | 0.318 | -0.175 | 0.039 | 6.12e-06 |
| 12:11266828 | rs77096743 | C/A | 0.318 | -0.175 | 0.039 | 6.12e-06 |
| 12:11271711 | rs68186227 | C/T | 0.318 | -0.175 | 0.039 | 6.12e-06 |
| 12:11216751 | rs67861347 | G/A | 0.329 | -0.172 | 0.038 | 6.18e-06 |
| 12:11165446 | rs4763613 | T/C | 0.331 | -0.170 | 0.038 | 6.53e-06 |
| 12:11154236 | rs10772412 | C/T | 0.327 | -0.171 | 0.038 | 6.69e-06 |
| 12:11149532 | rs1450839 | G/A | 0.331 | -0.169 | 0.038 | 6.82e-06 |
| 12:11149711 | rs10845279 | A/C | 0.331 | -0.169 | 0.038 | 6.82e-06 |
| 12:11149720 | rs10845280 | G/A | 0.331 | -0.169 | 0.038 | 6.82e-06 |
| 12:11149769 | rs10845281 | C/T | 0.331 | -0.169 | 0.038 | 6.82e-06 |
| 12:11150033 | rs12226919 | T/G | 0.331 | -0.169 | 0.038 | 6.82e-06 |
| 12:11150046 | rs12226920 | T/G | 0.331 | -0.169 | 0.038 | 6.82e-06 |
| 12:11150214 | rs11054142 | A/G | 0.331 | -0.169 | 0.038 | 6.82e-06 |
| 12:11150319 | rs11054143 | C/T | 0.331 | -0.169 | 0.038 | 6.82e-06 |
| 12:11150884 | rs7301234 | A/G | 0.331 | -0.169 | 0.038 | 6.82e-06 |
| 12:11150969 | rs7135941 | C/T | 0.331 | -0.169 | 0.038 | 6.82e-06 |
| 12:11151003 | rs7301364 | A/G | 0.331 | -0.169 | 0.038 | 6.82e-06 |
| 12:11151213 | rs7301713 | A/T | 0.331 | -0.169 | 0.038 | 6.82e-06 |
| 12:11151826 | rs10845282 | A/G | 0.331 | -0.169 | 0.038 | 6.82e-06 |
| 12:11152029 | rs11054144 | T/C | 0.331 | -0.169 | 0.038 | 6.82e-06 |
| 12:11152200 | rs11054145 | T/C | 0.331 | -0.169 | 0.038 | 6.82e-06 |
| 12:11152350 | rs11054146 | A/G | 0.331 | -0.169 | 0.038 | 6.82e-06 |
| 12:11152775 | rs2060702 | T/C | 0.331 | -0.169 | 0.038 | 6.82e-06 |
| 12:11153206 | rs1450840 | C/A | 0.331 | -0.169 | 0.038 | 6.82e-06 |
| 12:11153547 | rs11054147 | T/C | 0.331 | -0.169 | 0.038 | 6.82e-06 |
| 12:11154906 | rs12321023 | A/G | 0.331 | -0.169 | 0.038 | 6.82e-06 |
| 12:11156123 | rs10772413 | T/C | 0.331 | -0.169 | 0.038 | 6.82e-06 |
| 12:11157120 | rs7138834 | G/A | 0.331 | -0.169 | 0.038 | 6.82e-06 |
| 12:11158299 | rs10845284 | T/C | 0.331 | -0.169 | 0.038 | 6.82e-06 |
| 12:11158390 | rs10845285 | A/G | 0.331 | -0.169 | 0.038 | 6.82e-06 |
| 12:11159050 | rs10845286 | T/C | 0.331 | -0.169 | 0.038 | 6.82e-06 |
| 12:11159135 | rs10845287 | G/C | 0.331 | -0.169 | 0.038 | 6.82e-06 |
| 12:11159512 | rs11054152 | G/C | 0.331 | -0.169 | 0.038 | 6.82e-06 |
| 12:11159693 | rs11054154 | T/C | 0.331 | -0.169 | 0.038 | 6.82e-06 |
| 12:11160459 | rs10772415 | T/A | 0.331 | -0.169 | 0.038 | 6.82e-06 |
| 12:11160740 | rs4298989 | T/C | 0.331 | -0.169 | 0.038 | 6.82e-06 |
| 12:11160840 | rs1450841 | A/G | 0.331 | -0.169 | 0.038 | 6.82e-06 |
| 12:11161343 | rs7133669 | G/A | 0.331 | -0.169 | 0.038 | 6.82e-06 |
| 12:11161448 | rs36104587 | A/G | 0.331 | -0.169 | 0.038 | 6.82e-06 |
| 12:11161496 | rs35653945 | G/T | 0.331 | -0.169 | 0.038 | 6.82e-06 |
| 12:11161838 | rs28569398 | A/G | 0.331 | -0.169 | 0.038 | 6.82e-06 |
| 12:11161936 | rs28654530 | C/T | 0.331 | -0.169 | 0.038 | 6.82e-06 |
| 12:11161976 | rs28498385 | C/T | 0.331 | -0.169 | 0.038 | 6.82e-06 |
| 12:11162014 | rs28630880 | C/T | 0.331 | -0.169 | 0.038 | 6.82e-06 |
| 12:11162131 | rs11054156 | G/A | 0.331 | -0.169 | 0.038 | 6.82e-06 |
| 12:11162140 | rs11054157 | A/G | 0.331 | -0.169 | 0.038 | 6.82e-06 |
| 12:11162287 | rs11054158 | T/C | 0.331 | -0.169 | 0.038 | 6.82e-06 |
| 12:11162442 | rs11054159 | A/C | 0.331 | -0.169 | 0.038 | 6.82e-06 |
| 12:11162533 | rs11054160 | C/T | 0.331 | -0.169 | 0.038 | 6.82e-06 |
| 12:11162679 | rs11054161 | A/C | 0.331 | -0.169 | 0.038 | 6.82e-06 |
| 12:11162785 | rs11054162 | A/G | 0.331 | -0.169 | 0.038 | 6.82e-06 |
| 12:11162790 | rs11054163 | A/G | 0.331 | -0.169 | 0.038 | 6.82e-06 |
| 12:11162991 | rs4763606 | G/A | 0.331 | -0.169 | 0.038 | 6.82e-06 |
| 12:11163012 | rs4763607 | C/T | 0.331 | -0.169 | 0.038 | 6.82e-06 |
| 12:11163058 | rs4763608 | C/T | 0.331 | -0.169 | 0.038 | 6.82e-06 |
