# Supplementary material for: Bivariate genome-wide association analysis strengthens the role of bitter receptor clusters on chromosomes 7 and 12 in human bitter taste
Source: BMC Genomics. 2018 Sep 17;19:678. doi: 10.1186/s12864-018-5058-2 (PMC6142396; doi:10.1186/s12864-018-5058-2)
Supplement: Supplementary file 4 — Table S4. Top 100 SNPs on chromosome 7 associated with the perceived intensity of denatonium benzoate (DB) from the bivariate analysis of DB and quinine. P_univaraite_DB is the P-value from the univariate analysis of DB. P_univariate DB_adjQ is the P-value from the univariate analysis of DB adjusted for the quinine score. P_bivariate DB_Q is the P-value from the bivariate analysis of DB and quinine. (DOCX 218 kb) [file 12864_2018_5058_MOESM4_ESM.docx]

**Table S4. Top 100 SNPs on chromosome 7 associated with the perceived intensity of denatonium benzoate (DB) from the bivariate analysis of DB and quinine.** P_univaraite_DB is the P-value from the univariate analysis of DB. P_univariate DB_adjQ is the P-value from the univariate analysis of DB adjusted for the quinine score. P_bivariate DB_Q is the P-value from the bivariate analysis of DB and quinine.

| **Chr:Position** | **SNP** | **A1/A2** | **MAF** | **Beta**  **Univariate_DB** | **SE**  **Univariate_DB** | **P_univariate**  **DB** | **P_univariate**  **DB_adjQ** | **P_bivariate**  **DB_Q** |
| --- | --- | --- | --- | --- | --- | --- | --- | --- |
| 7:141398707 | rs10261515 | G/A | 0.491 | -0.136 | 0.037 | 2.54e-04 | 1.94e-08 | 3.15e-08 |
| 7:141428042 | rs2366501 | T/C | 0.498 | 0.118 | 0.037 | 1.49e-03 | 2.00e-07 | 1.90e-07 |
| 7:141416846 | rs6964456 | T/C | 0.499 | -0.120 | 0.037 | 1.18e-03 | 1.74e-07 | 1.92e-07 |
| 7:141419499 | rs12672225 | G/T | 0.496 | 0.127 | 0.037 | 5.63e-04 | 1.17e-07 | 1.96e-07 |
| 7:141421245 | rs12113603 | G/A | 0.5 | 0.118 | 0.037 | 1.38e-03 | 2.10e-07 | 2.28e-07 |
| 7:141421842 | rs4726471 | G/A | 0.5 | -0.118 | 0.037 | 1.38e-03 | 2.10e-07 | 2.28e-07 |
| 7:141421101 | rs6948307 | T/C | 0.5 | -0.118 | 0.037 | 1.38e-03 | 2.10e-07 | 2.28e-07 |
| 7:141425690 | rs34184650 | T/C | 0.499 | 0.117 | 0.037 | 1.58e-03 | 2.48e-07 | 2.50e-07 |
| 7:141421031 | rs6947065 | T/G | 0.5 | -0.119 | 0.037 | 1.26e-03 | 2.13e-07 | 2.52e-07 |
| 7:141420264 | rs10257653 | T/C | 0.499 | 0.119 | 0.037 | 1.25e-03 | 2.26e-07 | 2.74e-07 |
| 7:141418573 | rs10262864 | G/T | 0.499 | 0.119 | 0.037 | 1.24e-03 | 2.35e-07 | 2.85e-07 |
| 7:141415102 | rs9942597 | T/C | 0.5 | -0.119 | 0.037 | 1.25e-03 | 2.45e-07 | 2.89e-07 |
| 7:141415514 | rs9942694 | G/A | 0.5 | 0.119 | 0.037 | 1.25e-03 | 2.45e-07 | 2.89e-07 |
| 7:141421623 | rs17162425 | A/C | 0.499 | 0.116 | 0.037 | 1.62e-03 | 2.82e-07 | 2.94e-07 |
| 7:141420390 | rs6966981 | T/C | 0.496 | 0.124 | 0.037 | 7.32e-04 | 1.83e-07 | 3.06e-07 |
| 7:141420707 | rs6971275 | T/C | 0.496 | 0.124 | 0.037 | 7.43e-04 | 1.92e-07 | 3.25e-07 |
| 7:141420768 | rs6967301 | A/G | 0.496 | 0.124 | 0.037 | 7.45e-04 | 1.93e-07 | 3.26e-07 |
| 7:141389640 | rs7791469 | A/G | 0.487 | -0.115 | 0.036 | 1.60e-03 | 2.71e-07 | 3.26e-07 |
| 7:141414317 | rs2072180 | A/G | 0.496 | 0.123 | 0.037 | 8.11e-04 | 2.11e-07 | 3.30e-07 |
| 7:141422153 | rs4726472 | C/T | 0.496 | 0.120 | 0.037 | 1.10e-03 | 2.53e-07 | 3.62e-07 |
| 7:141417484 | rs4726470 | T/A | 0.496 | 0.121 | 0.037 | 9.96e-04 | 2.74e-07 | 4.27e-07 |
| 7:141433634 | rs1008318 | T/G | 0.495 | 0.119 | 0.037 | 1.26e-03 | 3.30e-07 | 4.52e-07 |
| 7:141601523 | rs1285900 | C/A | 0.471 | 0.092 | 0.037 | 1.32e-02 | 1.92e-06 | 5.17e-07 |
| 7:141429767 | rs6464452 | A/G | 0.433 | 0.115 | 0.037 | 2.05e-03 | 4.52e-07 | 5.29e-07 |
| 7:141543711 | rs12670179 | C/T | 0.469 | 0.099 | 0.038 | 8.57e-03 | 1.67e-06 | 6.21e-07 |
| 7:141601043 | rs1285899 | T/A | 0.471 | 0.085 | 0.037 | 2.09e-02 | 3.51e-06 | 6.95e-07 |
| 7:141435436 | rs12533304 | C/T | 0.496 | 0.100 | 0.037 | 7.95e-03 | 1.63e-06 | 7.00e-07 |
| 7:141501943 | rs17524275 | C/G | 0.486 | 0.096 | 0.037 | 9.41e-03 | 1.94e-06 | 7.17e-07 |
| 7:141530918 | rs6969093 | A/G | 0.478 | 0.104 | 0.037 | 5.29e-03 | 1.27e-06 | 7.33e-07 |
| 7:141529611 | rs4530955 | T/A | 0.478 | 0.107 | 0.037 | 4.21e-03 | 1.11e-06 | 7.38e-07 |
| 7:141530176 | rs7780596 | T/A | 0.478 | 0.104 | 0.037 | 5.34e-03 | 1.29e-06 | 7.41e-07 |
| 7:141532934 | rs12703410 | A/G | 0.477 | 0.102 | 0.037 | 5.83e-03 | 1.45e-06 | 7.92e-07 |
| 7:141483590 | rs12530637 | A/G | 0.397 | 0.086 | 0.039 | 2.64e-02 | 4.97e-06 | 8.25e-07 |
| 7:141505318 | rs62477743 | G/T | 0.486 | 0.094 | 0.037 | 1.10e-02 | 2.42e-06 | 8.41e-07 |
| 7:141504196 | rs11766522 | G/A | 0.486 | 0.094 | 0.037 | 1.11e-02 | 2.45e-06 | 8.53e-07 |
| 7:141532989 | rs12703411 | A/C | 0.477 | 0.104 | 0.037 | 5.29e-03 | 1.47e-06 | 8.65e-07 |
| 7:141519959 | rs1074968 | G/C | 0.478 | 0.100 | 0.037 | 7.44e-03 | 1.81e-06 | 8.97e-07 |
| 7:141592840 | rs1285954 | A/G | 0.471 | 0.085 | 0.037 | 2.19e-02 | 4.28e-06 | 9.24e-07 |
| 7:141381467 | rs12703403 | G/C | 0.45 | -0.124 | 0.037 | 8.37e-04 | 3.92e-07 | 9.29e-07 |
| 7:141517831 | rs11770781 | C/T | 0.478 | 0.101 | 0.037 | 6.69e-03 | 1.78e-06 | 9.57e-07 |
| 7:141517511 | rs2214839 | C/T | 0.478 | 0.101 | 0.037 | 6.69e-03 | 1.78e-06 | 9.57e-07 |
| 7:141465363 | rs6962760 | C/T | 0.491 | 0.103 | 0.037 | 5.10e-03 | 1.47e-06 | 1.01e-06 |
| 7:141589691 | rs1285956 | G/A | 0.471 | 0.086 | 0.037 | 1.98e-02 | 4.20e-06 | 1.05e-06 |
| 7:141495604 | rs1859646 | A/G | 0.486 | 0.101 | 0.037 | 6.07e-03 | 1.76e-06 | 1.05e-06 |
| 7:141398942 | rs13231650 | T/C | 0.433 | 0.114 | 0.037 | 1.81e-03 | 6.46e-07 | 1.08e-06 |
| 7:141497070 | rs34378880 | A/G | 0.485 | 0.098 | 0.037 | 8.12e-03 | 2.20e-06 | 1.10e-06 |
| 7:141412310 | rs1476640 | T/C | 0.434 | 0.104 | 0.037 | 4.88e-03 | 1.37e-06 | 1.12e-06 |
| 7:141501747 | rs12703408 | G/C | 0.483 | 0.100 | 0.037 | 6.96e-03 | 2.06e-06 | 1.17e-06 |
| 7:141501672 | rs17464668 | A/G | 0.483 | 0.100 | 0.037 | 6.96e-03 | 2.06e-06 | 1.17e-06 |
| 7:141525153 | rs7791243 | T/A | 0.478 | 0.099 | 0.037 | 7.78e-03 | 2.26e-06 | 1.19e-06 |
| 7:141626494 | rs1285931 | C/G | 0.451 | 0.079 | 0.037 | 3.42e-02 | 8.14e-06 | 1.24e-06 |
| 7:141591345 | rs1285955 | A/G | 0.47 | 0.084 | 0.037 | 2.42e-02 | 5.66e-06 | 1.26e-06 |
| 7:141544787 | rs892355 | C/T | 0.476 | 0.095 | 0.037 | 1.02e-02 | 3.03e-06 | 1.28e-06 |
| 7:141524776 | rs6464454 | A/G | 0.478 | 0.100 | 0.037 | 7.06e-03 | 2.28e-06 | 1.30e-06 |
| 7:141498467 | rs11762219 | C/T | 0.483 | 0.102 | 0.037 | 5.89e-03 | 1.97e-06 | 1.31e-06 |
| 7:141530057 | rs4636113 | A/T | 0.474 | 0.106 | 0.037 | 4.44e-03 | 1.61e-06 | 1.33e-06 |
| 7:141475161 | rs35046848 | A/G | 0.399 | 0.089 | 0.038 | 2.03e-02 | 4.87e-06 | 1.37e-06 |
| 7:141504604 | rs11763806 | T/C | 0.483 | 0.098 | 0.037 | 8.21e-03 | 2.58e-06 | 1.38e-06 |
| 7:141503999 | rs58726075 | A/G | 0.483 | 0.098 | 0.037 | 8.21e-03 | 2.58e-06 | 1.38e-06 |
| 7:141502792 | rs11766169 | C/T | 0.483 | 0.098 | 0.037 | 8.11e-03 | 2.57e-06 | 1.39e-06 |
| 7:141578863 | rs1830211 | C/T | 0.47 | 0.093 | 0.037 | 1.20e-02 | 3.44e-06 | 1.44e-06 |
| 7:141607214 | rs1799658 | G/A | 0.471 | 0.082 | 0.037 | 2.69e-02 | 6.86e-06 | 1.46e-06 |
| 7:141504298 | rs11769672 | A/G | 0.483 | 0.097 | 0.037 | 8.55e-03 | 2.79e-06 | 1.49e-06 |
| 7:141496256 | rs9648785 | C/T | 0.483 | 0.103 | 0.037 | 5.25e-03 | 1.99e-06 | 1.56e-06 |
| 7:141540548 | rs12533399 | C/T | 0.476 | 0.096 | 0.037 | 9.38e-03 | 3.28e-06 | 1.63e-06 |
| 7:141464765 | rs2270009 | T/C | 0.486 | 0.104 | 0.037 | 4.45e-03 | 1.80e-06 | 1.69e-06 |
| 7:141439907 | rs6464453 | C/G | 0.494 | 0.098 | 0.037 | 8.47e-03 | 2.79e-06 | 1.72e-06 |
| 7:141468253 | rs12667295 | T/C | 0.49 | 0.100 | 0.037 | 6.32e-03 | 2.43e-06 | 1.74e-06 |
| 7:141467838 | rs974008 | G/A | 0.49 | 0.100 | 0.037 | 6.32e-03 | 2.43e-06 | 1.74e-06 |
| 7:141486321 | rs11773137 | G/A | 0.49 | 0.093 | 0.037 | 1.13e-02 | 3.83e-06 | 1.78e-06 |
| 7:141540492 | rs12532841 | G/A | 0.476 | 0.097 | 0.037 | 8.54e-03 | 3.31e-06 | 1.79e-06 |
| 7:141537735 | rs12669721 | G/T | 0.476 | 0.097 | 0.037 | 8.54e-03 | 3.31e-06 | 1.79e-06 |
| 7:141538865 | rs12703412 | G/A | 0.476 | 0.097 | 0.037 | 8.54e-03 | 3.31e-06 | 1.79e-06 |
| 7:141442213 | rs2301924 | A/G | 0.495 | 0.094 | 0.037 | 1.21e-02 | 3.77e-06 | 1.81e-06 |
| 7:141407716 | rs6963959 | A/C | 0.431 | 0.106 | 0.037 | 4.19e-03 | 1.64e-06 | 1.82e-06 |
| 7:141518505 | rs34706333 | C/A | 0.474 | 0.101 | 0.037 | 6.60e-03 | 2.55e-06 | 1.83e-06 |
| 7:141512088 | rs13223389 | A/C | 0.474 | 0.101 | 0.037 | 6.76e-03 | 2.59e-06 | 1.83e-06 |
| 7:141518836 | rs12534862 | G/A | 0.474 | 0.101 | 0.037 | 6.67e-03 | 2.58e-06 | 1.84e-06 |
| 7:141514009 | rs34285424 | C/T | 0.474 | 0.101 | 0.037 | 6.74e-03 | 2.60e-06 | 1.84e-06 |
| 7:141514595 | rs7779209 | A/G | 0.474 | 0.101 | 0.037 | 6.71e-03 | 2.60e-06 | 1.85e-06 |
| 7:141565938 | rs1918301 | C/T | 0.47 | 0.091 | 0.037 | 1.42e-02 | 4.79e-06 | 1.85e-06 |
| 7:141407424 | rs4726468 | T/C | 0.43 | 0.107 | 0.037 | 3.84e-03 | 1.59e-06 | 1.94e-06 |
| 7:141442652 | rs2013816 | G/A | 0.495 | 0.095 | 0.037 | 1.11e-02 | 3.82e-06 | 2.01e-06 |
| 7:141614005 | rs1285912 | G/A | 0.471 | 0.078 | 0.037 | 3.56e-02 | 1.11e-05 | 2.14e-06 |
| 7:141561184 | rs1655265 | C/T | 0.469 | 0.090 | 0.037 | 1.52e-02 | 5.63e-06 | 2.16e-06 |
| 7:141558725 | rs2436718 | G/A | 0.469 | 0.090 | 0.037 | 1.52e-02 | 5.63e-06 | 2.16e-06 |
| 7:141561751 | rs2695133 | G/A | 0.469 | 0.090 | 0.037 | 1.52e-02 | 5.63e-06 | 2.16e-06 |
| 7:141466179 | rs62476658 | A/G | 0.487 | 0.105 | 0.037 | 4.26e-03 | 2.14e-06 | 2.20e-06 |
| 7:141564780 | rs1285896 | T/C | 0.469 | 0.088 | 0.037 | 1.75e-02 | 6.43e-06 | 2.28e-06 |
| 7:141524822 | rs7806962 | A/G | 0.475 | 0.102 | 0.037 | 5.91e-03 | 2.82e-06 | 2.32e-06 |
| 7:141480936 | rs17464086 | A/G | 0.489 | 0.097 | 0.036 | 7.66e-03 | 3.53e-06 | 2.35e-06 |
| 7:141478800 | rs2234002 | A/G | 0.489 | 0.097 | 0.036 | 7.66e-03 | 3.53e-06 | 2.35e-06 |
| 7:141444414 | rs17462840 | C/G | 0.494 | 0.092 | 0.037 | 1.36e-02 | 4.96e-06 | 2.35e-06 |
| 7:141526913 | rs7804754 | C/T | 0.479 | 0.098 | 0.037 | 8.24e-03 | 3.71e-06 | 2.35e-06 |
| 7:141469679 | rs2023998 | A/G | 0.487 | 0.103 | 0.037 | 5.01e-03 | 2.52e-06 | 2.40e-06 |
| 7:141572858 | rs1285895 | A/G | 0.47 | 0.088 | 0.037 | 1.77e-02 | 6.90e-06 | 2.43e-06 |
| 7:141489911 | rs2234007 | A/G | 0.489 | 0.089 | 0.037 | 1.50e-02 | 5.86e-06 | 2.44e-06 |
| 7:141488985 | rs10952507 | A/G | 0.489 | 0.089 | 0.037 | 1.50e-02 | 5.86e-06 | 2.44e-06 |
| 7:141489866 | rs2234006 | T/C | 0.489 | 0.089 | 0.037 | 1.50e-02 | 5.86e-06 | 2.44e-06 |
| 7:141475054 | rs4535645 | C/T | 0.49 | 0.098 | 0.037 | 7.60e-03 | 3.58e-06 | 2.46e-06 |
