# Supplementary material for: Bivariate genome-wide association analysis strengthens the role of bitter receptor clusters on chromosomes 7 and 12 in human bitter taste
Source: BMC Genomics. 2018 Sep 17;19:678. doi: 10.1186/s12864-018-5058-2 (PMC6142396; doi:10.1186/s12864-018-5058-2)
Supplement: Supplementary file 5 — Table S5. Top 100 SNPs on chromosome 7 associated with the perceived intensity of PROP solution. (DOCX 175 kb) [file 12864_2018_5058_MOESM5_ESM.docx]

**Table S5. Top 100 SNPs on chromosome 7 associated with the perceived intensity of PROP solution.**

| **Chr:Position** | **SNP** | **A1/A2** | **MAF** | **Beta** | **SE** | **P** |
| --- | --- | --- | --- | --- | --- | --- |
| 7:141672604 | rs10246939 | C/T | 0.443 | 0.968 | 0.028 | 2.80e-199 |
| 7:141672705 | rs1726866 | G/A | 0.443 | 0.965 | 0.028 | 5.62e-198 |
| 7:141662394 | rs2695135 | T/C | 0.282 | 0.756 | 0.040 | 1.80e-73 |
| 7:141658390 | rs2436717 | T/C | 0.278 | 0.741 | 0.039 | 2.24e-73 |
| 7:141661450 | rs6962383 | C/A | 0.28 | 0.749 | 0.040 | 1.82e-72 |
| 7:141660700 | rs1726867 | A/G | 0.281 | 0.740 | 0.039 | 7.28e-72 |
| 7:141653637 | rs7794708 | T/C | 0.275 | 0.696 | 0.039 | 1.81e-66 |
| 7:141639975 | rs1285944 | C/T | 0.282 | 0.687 | 0.039 | 8.13e-65 |
| 7:141636563 | rs1285950 | C/A | 0.282 | 0.687 | 0.039 | 1.38e-64 |
| 7:141647022 | rs1285968 | A/G | 0.279 | 0.683 | 0.039 | 1.17e-63 |
| 7:141613205 | rs13240104 | G/A | 0.282 | 0.677 | 0.039 | 4.09e-63 |
| 7:141613248 | rs34588922 | C/T | 0.282 | 0.677 | 0.039 | 4.09e-63 |
| 7:141612717 | rs6976028 | T/C | 0.282 | 0.676 | 0.039 | 5.09e-63 |
| 7:141546847 | rs12703413 | A/G | 0.278 | 0.676 | 0.039 | 5.28e-62 |
| 7:141544200 | rs9640205 | G/C | 0.273 | 0.684 | 0.040 | 5.41e-62 |
| 7:141551958 | rs34894166 | C/T | 0.279 | 0.674 | 0.039 | 1.12e-61 |
| 7:141564646 | rs62475469 | A/G | 0.279 | 0.674 | 0.039 | 1.70e-61 |
| 7:141560990 | rs60165685 | A/G | 0.278 | 0.674 | 0.039 | 2.22e-61 |
| 7:141586441 | rs6955562 | G/C | 0.276 | 0.676 | 0.039 | 2.43e-61 |
| 7:141556519 | rs58093678 | G/C | 0.278 | 0.673 | 0.039 | 3.38e-61 |
| 7:141560655 | rs17133534 | T/G | 0.279 | 0.673 | 0.039 | 3.60e-61 |
| 7:141562424 | rs13232651 | T/C | 0.279 | 0.673 | 0.039 | 3.60e-61 |
| 7:141533757 | rs35647444 | T/G | 0.279 | 0.672 | 0.039 | 4.94e-61 |
| 7:141633062 | rs1527309 | T/C | 0.28 | 0.671 | 0.039 | 5.11e-61 |
| 7:141531140 | rs6969430 | A/G | 0.28 | 0.667 | 0.039 | 6.91e-60 |
| 7:141590705 | rs10808016 | G/T | 0.281 | 0.663 | 0.039 | 9.97e-60 |
| 7:141532187 | rs10464444 | A/G | 0.28 | 0.666 | 0.039 | 1.01e-59 |
| 7:141588426 | rs35634557 | C/T | 0.283 | 0.661 | 0.039 | 1.31e-59 |
| 7:141565357 | rs6957037 | G/A | 0.282 | 0.660 | 0.039 | 3.35e-59 |
| 7:141588055 | rs873818 | T/G | 0.281 | 0.662 | 0.039 | 3.77e-59 |
| 7:141543098 | rs9640357 | T/C | 0.282 | 0.659 | 0.039 | 3.94e-59 |
| 7:141543810 | rs35836873 | G/A | 0.282 | 0.659 | 0.039 | 3.94e-59 |
| 7:141543882 | rs9640358 | A/G | 0.282 | 0.659 | 0.039 | 3.94e-59 |
| 7:141544100 | rs9640204 | G/A | 0.28 | 0.660 | 0.039 | 8.05e-59 |
| 7:141537968 | rs11765575 | G/A | 0.282 | 0.657 | 0.039 | 8.48e-59 |
| 7:141544199 | rs9640359 | A/C | 0.275 | 0.666 | 0.040 | 1.04e-58 |
| 7:141584184 | rs13235900 | G/A | 0.279 | 0.659 | 0.039 | 2.58e-58 |
| 7:141585166 | rs1980369 | G/T | 0.279 | 0.659 | 0.039 | 2.58e-58 |
| 7:141574911 | rs12668089 | T/C | 0.281 | 0.655 | 0.039 | 2.78e-58 |
| 7:141575800 | rs12668693 | T/C | 0.281 | 0.655 | 0.039 | 2.78e-58 |
| 7:141577186 | rs2082551 | G/A | 0.281 | 0.655 | 0.039 | 2.78e-58 |
| 7:141569606 | rs6959360 | A/C | 0.281 | 0.654 | 0.039 | 3.40e-58 |
| 7:141573055 | rs12534927 | C/T | 0.281 | 0.654 | 0.039 | 3.77e-58 |
| 7:141567569 | rs2163953 | C/T | 0.281 | 0.653 | 0.039 | 4.89e-58 |
| 7:141661585 | rs10435196 | T/A | 0.248 | 0.686 | 0.042 | 6.28e-57 |
| 7:141658886 | rs67596995 | G/A | 0.25 | 0.678 | 0.041 | 7.06e-57 |
| 7:141662547 | rs12531134 | C/T | 0.249 | 0.687 | 0.042 | 1.06e-56 |
| 7:141656487 | rs11762634 | A/G | 0.25 | 0.661 | 0.041 | 1.16e-55 |
| 7:141657465 | rs13235385 | T/C | 0.246 | 0.666 | 0.041 | 3.47e-55 |
| 7:141654892 | rs2570407 | C/A | 0.251 | 0.658 | 0.041 | 3.97e-55 |
| 7:141637810 | rs1594777 | G/A | 0.253 | 0.642 | 0.041 | 1.04e-52 |
| 7:141638297 | rs12531781 | T/C | 0.253 | 0.642 | 0.041 | 1.04e-52 |
| 7:141638429 | rs13227402 | T/C | 0.253 | 0.642 | 0.041 | 1.04e-52 |
| 7:141642285 | rs1594776 | T/C | 0.248 | 0.636 | 0.041 | 3.02e-51 |
| 7:141639215 | rs13237944 | A/C | 0.249 | 0.635 | 0.041 | 4.63e-51 |
| 7:141646430 | rs2293460 | T/C | 0.246 | 0.632 | 0.041 | 4.96e-50 |
| 7:141646434 | rs2293461 | G/A | 0.246 | 0.632 | 0.041 | 4.96e-50 |
| 7:141630267 | rs12539499 | C/T | 0.251 | 0.626 | 0.041 | 2.83e-49 |
| 7:141593434 | rs12538701 | T/C | 0.252 | 0.622 | 0.041 | 8.30e-49 |
| 7:141544734 | rs892354 | T/C | 0.251 | 0.622 | 0.041 | 8.72e-49 |
| 7:141544095 | rs9640203 | G/A | 0.25 | 0.624 | 0.041 | 1.07e-48 |
| 7:141628704 | rs11770855 | C/T | 0.25 | 0.622 | 0.041 | 1.32e-48 |
| 7:141550780 | rs13236432 | C/G | 0.251 | 0.621 | 0.041 | 1.47e-48 |
| 7:141590684 | rs10952509 | C/A | 0.25 | 0.622 | 0.041 | 1.51e-48 |
| 7:141627899 | rs13222726 | G/A | 0.25 | 0.619 | 0.041 | 2.20e-48 |
| 7:141602476 | rs11765106 | G/A | 0.252 | 0.616 | 0.041 | 4.46e-48 |
| 7:141609424 | rs7802271 | A/T | 0.254 | 0.610 | 0.041 | 7.34e-48 |
| 7:141609758 | rs7782886 | A/G | 0.254 | 0.610 | 0.041 | 7.34e-48 |
| 7:141610572 | rs35412929 | C/T | 0.254 | 0.610 | 0.041 | 7.34e-48 |
| 7:141610891 | rs994808 | C/A | 0.254 | 0.610 | 0.041 | 7.34e-48 |
| 7:141611285 | rs994809 | C/T | 0.254 | 0.610 | 0.041 | 7.34e-48 |
| 7:141611392 | rs7808421 | G/A | 0.254 | 0.610 | 0.041 | 7.34e-48 |
| 7:141611499 | rs7789123 | C/G | 0.254 | 0.610 | 0.041 | 7.34e-48 |
| 7:141612116 | rs11767119 | A/G | 0.254 | 0.610 | 0.041 | 7.34e-48 |
| 7:141612621 | rs11767947 | A/G | 0.254 | 0.610 | 0.041 | 7.34e-48 |
| 7:141614110 | rs11769089 | A/G | 0.253 | 0.611 | 0.041 | 8.40e-48 |
| 7:141614190 | rs11765974 | G/A | 0.253 | 0.611 | 0.041 | 8.40e-48 |
| 7:141605899 | rs7786202 | C/T | 0.253 | 0.613 | 0.041 | 1.11e-47 |
| 7:141531917 | rs34726057 | C/T | 0.252 | 0.611 | 0.041 | 1.39e-46 |
| 7:141579215 | rs10952508 | G/T | 0.25 | 0.607 | 0.041 | 1.99e-46 |
| 7:141611955 | rs7785954 | G/A | 0.258 | 0.592 | 0.041 | 2.32e-45 |
| 7:141526020 | rs6967189 | C/T | 0.255 | 0.591 | 0.042 | 1.42e-43 |
| 7:141511858 | rs12703409 | C/T | 0.253 | 0.593 | 0.042 | 3.63e-42 |
| 7:141510353 | rs35010424 | T/C | 0.25 | 0.587 | 0.043 | 1.62e-40 |
| 7:141627149 | rs1285933 | G/A | 0.469 | 0.479 | 0.036 | 1.80e-38 |
| 7:141614005 | rs1285912 | G/A | 0.471 | 0.479 | 0.036 | 3.24e-38 |
| 7:141607214 | rs1799658 | G/A | 0.47 | 0.480 | 0.036 | 3.46e-38 |
| 7:141616506 | rs745162 | G/A | 0.471 | 0.478 | 0.036 | 3.92e-38 |
| 7:141615875 | rs1285914 | G/A | 0.471 | 0.478 | 0.036 | 5.03e-38 |
| 7:141475161 | rs35046848 | A/G | 0.399 | 0.497 | 0.038 | 5.08e-38 |
| 7:141615867 | rs1285913 | C/G | 0.471 | 0.478 | 0.036 | 5.33e-38 |
| 7:141591345 | rs1285955 | A/G | 0.47 | 0.477 | 0.036 | 1.20e-37 |
| 7:141589691 | rs1285956 | G/A | 0.471 | 0.477 | 0.036 | 1.26e-37 |
| 7:141429767 | rs6464452 | A/G | 0.432 | 0.482 | 0.037 | 1.64e-37 |
| 7:141592840 | rs1285954 | A/G | 0.47 | 0.476 | 0.036 | 1.65e-37 |
| 7:141601043 | rs1285899 | T/A | 0.471 | 0.476 | 0.036 | 1.74e-37 |
| 7:141601523 | rs1285900 | C/A | 0.471 | 0.475 | 0.036 | 1.99e-37 |
| 7:141563970 | rs1433594 | A/G | 0.466 | 0.476 | 0.036 | 2.04e-37 |
| 7:141368300 | rs12154227 | T/C | 0.424 | -0.483 | 0.037 | 2.50e-37 |
| 7:141549635 | rs34708913 | A/T | 0.465 | 0.475 | 0.036 | 3.14e-37 |
