# Supplementary material for: Bivariate genome-wide association analysis strengthens the role of bitter receptor clusters on chromosomes 7 and 12 in human bitter taste
Source: BMC Genomics. 2018 Sep 17;19:678. doi: 10.1186/s12864-018-5058-2 (PMC6142396; doi:10.1186/s12864-018-5058-2)
Supplement: Supplementary file 6 — Table S6. Top 100 SNPs on chromosome 7 associated with the perceived intensity of PROP paper. (DOCX 171 kb) [file 12864_2018_5058_MOESM6_ESM.docx]

**Table S6. Top 100 SNPs on chromosome 7 associated with the perceived intensity of PROP paper.**

| **Chr:Position** | **SNP** | **A1/A2** | **MAF** | **Beta** | **SE** | **P** |
| --- | --- | --- | --- | --- | --- | --- |
| 7:141672705 | rs1726866 | G/A | 0.441 | 0.535 | 0.032 | 3.38e-59 |
| 7:141672604 | rs10246939 | C/T | 0.441 | 0.534 | 0.032 | 5.40e-59 |
| 7:141658390 | rs2436717 | T/C | 0.278 | 0.461 | 0.038 | 4.37e-33 |
| 7:141660700 | rs1726867 | A/G | 0.281 | 0.457 | 0.038 | 8.77e-32 |
| 7:141661450 | rs6962383 | C/A | 0.28 | 0.458 | 0.039 | 1.73e-31 |
| 7:141662394 | rs2695135 | T/C | 0.282 | 0.459 | 0.039 | 2.41e-31 |
| 7:141653637 | rs7794708 | T/C | 0.276 | 0.433 | 0.037 | 4.68e-30 |
| 7:141639975 | rs1285944 | C/T | 0.283 | 0.427 | 0.037 | 2.87e-29 |
| 7:141636563 | rs1285950 | C/A | 0.283 | 0.427 | 0.037 | 3.32e-29 |
| 7:141647022 | rs1285968 | A/G | 0.279 | 0.424 | 0.037 | 9.69e-29 |
| 7:141613205 | rs13240104 | G/A | 0.282 | 0.415 | 0.037 | 5.34e-28 |
| 7:141613248 | rs34588922 | C/T | 0.282 | 0.415 | 0.037 | 5.34e-28 |
| 7:141612717 | rs6976028 | T/C | 0.283 | 0.413 | 0.037 | 9.20e-28 |
| 7:141633062 | rs1527309 | T/C | 0.282 | 0.413 | 0.038 | 2.61e-27 |
| 7:141586441 | rs6955562 | G/C | 0.276 | 0.409 | 0.038 | 1.68e-26 |
| 7:141546847 | rs12703413 | A/G | 0.279 | 0.407 | 0.038 | 1.96e-26 |
| 7:141551958 | rs34894166 | C/T | 0.279 | 0.406 | 0.038 | 2.32e-26 |
| 7:141533757 | rs35647444 | T/G | 0.279 | 0.405 | 0.038 | 2.89e-26 |
| 7:141590705 | rs10808016 | G/T | 0.282 | 0.404 | 0.038 | 3.00e-26 |
| 7:141531140 | rs6969430 | A/G | 0.28 | 0.406 | 0.038 | 3.11e-26 |
| 7:141532187 | rs10464444 | A/G | 0.28 | 0.406 | 0.038 | 3.40e-26 |
| 7:141588426 | rs35634557 | C/T | 0.283 | 0.403 | 0.038 | 3.44e-26 |
| 7:141564646 | rs62475469 | A/G | 0.28 | 0.405 | 0.038 | 3.46e-26 |
| 7:141556519 | rs58093678 | G/C | 0.279 | 0.405 | 0.038 | 3.53e-26 |
| 7:141560655 | rs17133534 | T/G | 0.279 | 0.405 | 0.038 | 3.63e-26 |
| 7:141562424 | rs13232651 | T/C | 0.279 | 0.405 | 0.038 | 3.63e-26 |
| 7:141560990 | rs60165685 | A/G | 0.279 | 0.405 | 0.038 | 4.13e-26 |
| 7:141544200 | rs9640205 | G/C | 0.273 | 0.408 | 0.038 | 6.69e-26 |
| 7:141544100 | rs9640204 | G/A | 0.279 | 0.401 | 0.038 | 1.22e-25 |
| 7:141588055 | rs873818 | T/G | 0.281 | 0.400 | 0.038 | 1.31e-25 |
| 7:141537968 | rs11765575 | G/A | 0.282 | 0.397 | 0.038 | 2.00e-25 |
| 7:141584184 | rs13235900 | G/A | 0.279 | 0.400 | 0.038 | 2.06e-25 |
| 7:141585166 | rs1980369 | G/T | 0.279 | 0.400 | 0.038 | 2.06e-25 |
| 7:141543098 | rs9640357 | T/C | 0.282 | 0.396 | 0.038 | 2.78e-25 |
| 7:141543810 | rs35836873 | G/A | 0.282 | 0.396 | 0.038 | 2.78e-25 |
| 7:141543882 | rs9640358 | A/G | 0.282 | 0.395 | 0.038 | 3.62e-25 |
| 7:141565357 | rs6957037 | G/A | 0.282 | 0.395 | 0.038 | 4.15e-25 |
| 7:141567569 | rs2163953 | C/T | 0.281 | 0.394 | 0.038 | 6.24e-25 |
| 7:141574911 | rs12668089 | T/C | 0.281 | 0.393 | 0.038 | 6.97e-25 |
| 7:141575800 | rs12668693 | T/C | 0.281 | 0.393 | 0.038 | 6.97e-25 |
| 7:141577186 | rs2082551 | G/A | 0.281 | 0.393 | 0.038 | 6.97e-25 |
| 7:141569606 | rs6959360 | A/C | 0.281 | 0.393 | 0.038 | 7.20e-25 |
| 7:141658886 | rs67596995 | G/A | 0.25 | 0.410 | 0.039 | 7.70e-25 |
| 7:141573055 | rs12534927 | C/T | 0.28 | 0.392 | 0.038 | 8.83e-25 |
| 7:141544199 | rs9640359 | A/C | 0.274 | 0.397 | 0.038 | 1.10e-24 |
| 7:141661585 | rs10435196 | T/A | 0.247 | 0.410 | 0.040 | 2.72e-24 |
| 7:141662547 | rs12531134 | C/T | 0.248 | 0.410 | 0.040 | 4.32e-24 |
| 7:141654892 | rs2570407 | C/A | 0.251 | 0.398 | 0.039 | 4.74e-24 |
| 7:141657465 | rs13235385 | T/C | 0.245 | 0.402 | 0.039 | 5.92e-24 |
| 7:141656487 | rs11762634 | A/G | 0.25 | 0.397 | 0.039 | 6.23e-24 |
| 7:141637810 | rs1594777 | G/A | 0.253 | 0.387 | 0.039 | 7.01e-23 |
| 7:141638297 | rs12531781 | T/C | 0.253 | 0.387 | 0.039 | 7.01e-23 |
| 7:141638429 | rs13227402 | T/C | 0.253 | 0.387 | 0.039 | 7.01e-23 |
| 7:141642285 | rs1594776 | T/C | 0.248 | 0.384 | 0.039 | 2.13e-22 |
| 7:141639215 | rs13237944 | A/C | 0.249 | 0.384 | 0.039 | 2.71e-22 |
| 7:141614110 | rs11769089 | A/G | 0.253 | 0.378 | 0.039 | 5.97e-22 |
| 7:141614190 | rs11765974 | G/A | 0.253 | 0.378 | 0.039 | 5.97e-22 |
| 7:141630267 | rs12539499 | C/T | 0.251 | 0.375 | 0.039 | 2.86e-21 |
| 7:141646430 | rs2293460 | T/C | 0.245 | 0.377 | 0.039 | 3.02e-21 |
| 7:141646434 | rs2293461 | G/A | 0.245 | 0.377 | 0.039 | 3.02e-21 |
| 7:141628704 | rs11770855 | C/T | 0.25 | 0.373 | 0.039 | 4.30e-21 |
| 7:141609424 | rs7802271 | A/T | 0.254 | 0.367 | 0.039 | 8.13e-21 |
| 7:141609758 | rs7782886 | A/G | 0.254 | 0.367 | 0.039 | 8.13e-21 |
| 7:141610572 | rs35412929 | C/T | 0.254 | 0.367 | 0.039 | 8.13e-21 |
| 7:141610891 | rs994808 | C/A | 0.254 | 0.367 | 0.039 | 8.13e-21 |
| 7:141611285 | rs994809 | C/T | 0.254 | 0.367 | 0.039 | 8.13e-21 |
| 7:141611392 | rs7808421 | G/A | 0.254 | 0.367 | 0.039 | 8.13e-21 |
| 7:141611499 | rs7789123 | C/G | 0.254 | 0.367 | 0.039 | 8.13e-21 |
| 7:141612116 | rs11767119 | A/G | 0.254 | 0.367 | 0.039 | 8.13e-21 |
| 7:141612621 | rs11767947 | A/G | 0.254 | 0.367 | 0.039 | 8.13e-21 |
| 7:141627899 | rs13222726 | G/A | 0.25 | 0.370 | 0.039 | 8.62e-21 |
| 7:141590684 | rs10952509 | C/A | 0.25 | 0.370 | 0.039 | 1.13e-20 |
| 7:141593434 | rs12538701 | T/C | 0.252 | 0.369 | 0.039 | 1.18e-20 |
| 7:141602476 | rs11765106 | G/A | 0.252 | 0.365 | 0.039 | 2.54e-20 |
| 7:141605899 | rs7786202 | C/T | 0.252 | 0.362 | 0.039 | 4.85e-20 |
| 7:141611955 | rs7785954 | G/A | 0.257 | 0.356 | 0.039 | 8.37e-20 |
| 7:141544095 | rs9640203 | G/A | 0.249 | 0.362 | 0.040 | 1.32e-19 |
| 7:141544734 | rs892354 | T/C | 0.251 | 0.355 | 0.039 | 3.56e-19 |
| 7:141550780 | rs13236432 | C/G | 0.251 | 0.355 | 0.039 | 3.84e-19 |
| 7:141531917 | rs34726057 | C/T | 0.253 | 0.354 | 0.039 | 5.57e-19 |
| 7:141579215 | rs10952508 | G/T | 0.25 | 0.353 | 0.039 | 6.28e-19 |
| 7:141526020 | rs6967189 | C/T | 0.256 | 0.349 | 0.039 | 2.23e-18 |
| 7:141511858 | rs12703409 | C/T | 0.254 | 0.343 | 0.040 | 3.23e-17 |
| 7:141510353 | rs35010424 | T/C | 0.252 | 0.337 | 0.041 | 1.93e-16 |
| 7:141616506 | rs745162 | G/A | 0.471 | 0.265 | 0.034 | 1.91e-14 |
| 7:141614005 | rs1285912 | G/A | 0.471 | 0.265 | 0.034 | 2.45e-14 |
| 7:141615875 | rs1285914 | G/A | 0.47 | 0.263 | 0.035 | 3.83e-14 |
| 7:141615867 | rs1285913 | C/G | 0.47 | 0.263 | 0.035 | 3.92e-14 |
| 7:141627149 | rs1285933 | G/A | 0.469 | 0.260 | 0.034 | 5.98e-14 |
| 7:141592840 | rs1285954 | A/G | 0.472 | 0.260 | 0.035 | 8.25e-14 |
| 7:141591345 | rs1285955 | A/G | 0.471 | 0.259 | 0.035 | 1.05e-13 |
| 7:141607214 | rs1799658 | G/A | 0.47 | 0.258 | 0.035 | 1.21e-13 |
| 7:141589691 | rs1285956 | G/A | 0.472 | 0.258 | 0.035 | 1.31e-13 |
| 7:141563970 | rs1433594 | A/G | 0.467 | 0.257 | 0.035 | 1.91e-13 |
| 7:141601043 | rs1285899 | T/A | 0.471 | 0.255 | 0.035 | 2.15e-13 |
| 7:141549635 | rs34708913 | A/T | 0.467 | 0.256 | 0.035 | 2.21e-13 |
| 7:141530057 | rs4636113 | A/T | 0.474 | 0.257 | 0.035 | 2.32e-13 |
| 7:141518505 | rs34706333 | C/A | 0.474 | 0.258 | 0.035 | 2.39e-13 |
| 7:141518836 | rs12534862 | G/A | 0.474 | 0.257 | 0.035 | 2.43e-13 |
| 7:141522086 | rs79390963 | G/A | 0.475 | 0.256 | 0.035 | 2.58e-13 |
