# Supplementary material for: Bivariate genome-wide association analysis strengthens the role of bitter receptor clusters on chromosomes 7 and 12 in human bitter taste
Source: BMC Genomics. 2018 Sep 17;19:678. doi: 10.1186/s12864-018-5058-2 (PMC6142396; doi:10.1186/s12864-018-5058-2)
Supplement: Supplementary file 7 — Table S7. Top 100 SNPs on chromosome 2 associated with the perceived intensity of PROP paper and their associations with PROP solution. (DOCX 183 kb) [file 12864_2018_5058_MOESM7_ESM.docx]

**Table S7. Top 100 SNPs on chromosome 2 associated with the perceived intensity of PROP paper and their associations with PROP solution.**

| **Chr:Position** | **SNP** | **A1/A2** | **MAF** | **Beta_paper** | **SE_paper** | **P_paper** | **P_solution** |
| --- | --- | --- | --- | --- | --- | --- | --- |
| 2:218218646 | rs6761655 | G/A | 0.186 | -0.246 | 0.044 | 2.69e-08 | 7.38e-04 |
| 2:218218695 | rs6736242 | A/G | 0.186 | -0.246 | 0.044 | 2.69e-08 | 7.38e-04 |
| 2:218219311 | rs7586502 | A/G | 0.142 | -0.259 | 0.049 | 1.31e-07 | 1.03e-03 |
| 2:218220180 | rs80312552 | G/A | 0.14 | -0.258 | 0.049 | 1.70e-07 | 9.43e-04 |
| 2:218219166 | rs6435978 | T/C | 0.142 | -0.256 | 0.049 | 1.85e-07 | 2.48e-03 |
| 2:218219074 | rs6435976 | T/C | 0.142 | -0.253 | 0.049 | 2.15e-07 | 2.34e-03 |
| 2:218219139 | rs6435977 | A/G | 0.143 | -0.254 | 0.049 | 2.18e-07 | 2.12e-03 |
| 2:218221469 | rs4674157 | A/G | 0.14 | -0.255 | 0.049 | 2.59e-07 | 1.07e-03 |
| 2:218220849 | rs4674155 | A/G | 0.139 | -0.252 | 0.049 | 3.60e-07 | 1.67e-03 |
| 2:218220860 | rs4674156 | T/C | 0.139 | -0.252 | 0.049 | 3.60e-07 | 1.67e-03 |
| 2:218218855 | rs6707253 | G/A | 0.142 | -0.248 | 0.049 | 3.77e-07 | 3.04e-03 |
| 2:218218774 | rs6707229 | C/A | 0.143 | -0.248 | 0.049 | 4.00e-07 | 3.09e-03 |
| 2:218188539 | rs13023129 | C/T | 0.232 | -0.211 | 0.042 | 4.99e-07 | 6.02e-03 |
| 2:218216220 | rs1863193 | C/T | 0.211 | -0.215 | 0.043 | 5.25e-07 | 7.78e-04 |
| 2:218214057 | rs13008830 | C/T | 0.211 | -0.214 | 0.043 | 6.69e-07 | 5.76e-04 |
| 2:218175472 | rs4141835 | A/G | 0.231 | -0.205 | 0.041 | 8.12e-07 | 5.93e-03 |
| 2:218200026 | rs13432162 | A/G | 0.229 | -0.206 | 0.042 | 1.14e-06 | 1.75e-03 |
| 2:218177694 | rs1863183 | T/C | 0.235 | -0.201 | 0.041 | 1.18e-06 | 1.09e-02 |
| 2:218197359 | rs16857324 | C/T | 0.231 | -0.204 | 0.042 | 1.47e-06 | 2.73e-03 |
| 2:218226173 | rs13417769 | G/A | 0.137 | -0.240 | 0.051 | 2.24e-06 | 5.42e-03 |
| 2:218228349 | rs7561131 | C/T | 0.205 | -0.203 | 0.043 | 3.31e-06 | 1.38e-03 |
| 2:218220232 | rs112802287 | G/A | 0.208 | -0.195 | 0.042 | 4.26e-06 | 3.89e-04 |
| 2:218222700 | rs4674158 | T/A | 0.209 | -0.193 | 0.042 | 5.50e-06 | 6.82e-04 |
| 2:218207688 | rs5028238 | G/A | 0.233 | -0.193 | 0.043 | 6.09e-06 | 1.20e-03 |
| 2:160387482 | rs34251858 | C/A | 0.094 | 0.258 | 0.059 | 1.31e-05 | 1.41e-02 |
| 2:112444296 | rs10186692 | G/T | 0.451 | 0.150 | 0.035 | 1.58e-05 | 2.82e-02 |
| 2:112443852 | rs10175681 | T/C | 0.449 | 0.148 | 0.034 | 1.83e-05 | 2.03e-02 |
| 2:218219697 | rs78832202 | G/A | 0.102 | -0.240 | 0.056 | 2.01e-05 | 8.90e-02 |
| 2:218219641 | rs79707432 | A/G | 0.102 | -0.239 | 0.056 | 2.02e-05 | 1.02e-01 |
| 2:218219226 | rs55848226 | A/G | 0.105 | -0.235 | 0.055 | 2.28e-05 | 1.37e-01 |
| 2:160340777 | rs35745662 | C/T | 0.102 | 0.245 | 0.058 | 2.49e-05 | 4.27e-02 |
| 2:160362019 | rs34081025 | A/T | 0.102 | 0.243 | 0.057 | 2.54e-05 | 3.12e-02 |
| 2:96777168 | rs2312955 | T/G | 0.34 | 0.151 | 0.036 | 2.70e-05 | 2.96e-01 |
| 2:160340014 | rs13017222 | A/G | 0.102 | 0.245 | 0.058 | 2.71e-05 | 4.22e-02 |
| 2:96780716 | rs2229169 | T/G | 0.34 | 0.151 | 0.036 | 2.74e-05 | 2.67e-01 |
| 2:96751395 | rs2140938 | C/T | 0.353 | 0.153 | 0.036 | 2.78e-05 | 5.76e-01 |
| 2:96774981 | rs7561198 | C/G | 0.34 | 0.151 | 0.036 | 2.81e-05 | 3.06e-01 |
| 2:96781986 | rs3111873 | G/C | 0.34 | 0.151 | 0.036 | 2.82e-05 | 2.79e-01 |
| 2:96784934 | rs2692894 | T/G | 0.34 | 0.151 | 0.036 | 2.82e-05 | 2.79e-01 |
| 2:105548062 | rs10496387 | T/C | 0.273 | -0.166 | 0.040 | 2.89e-05 | 2.94e-01 |
| 2:218163031 | rs74910011 | T/G | 0.119 | -0.223 | 0.053 | 3.04e-05 | 4.38e-01 |
| 2:96780122 | rs4907299 | T/G | 0.314 | 0.160 | 0.038 | 3.22e-05 | 1.68e-01 |
| 2:96931846 | rs2301707 | C/T | 0.322 | 0.155 | 0.037 | 3.29e-05 | 4.40e-01 |
| 2:105605488 | rs7574780 | C/A | 0.267 | -0.166 | 0.040 | 3.49e-05 | 4.04e-01 |
| 2:96831355 | rs1724125 | A/G | 0.34 | 0.150 | 0.036 | 3.53e-05 | 3.40e-01 |
| 2:160352948 | rs71423016 | T/C | 0.097 | 0.244 | 0.059 | 3.70e-05 | 5.67e-02 |
| 2:112445659 | rs6708131 | C/T | 0.448 | 0.143 | 0.035 | 3.78e-05 | 3.54e-02 |
| 2:105602952 | rs6543279 | G/A | 0.267 | -0.165 | 0.040 | 3.79e-05 | 4.11e-01 |
| 2:96794957 | rs2917662 | A/G | 0.34 | 0.148 | 0.036 | 3.81e-05 | 2.91e-01 |
| 2:96794982 | rs2969491 | T/C | 0.34 | 0.148 | 0.036 | 3.81e-05 | 2.91e-01 |
| 2:96787899 | rs1168965 | C/G | 0.34 | 0.148 | 0.036 | 4.18e-05 | 2.98e-01 |
| 2:105555869 | rs7595767 | G/T | 0.272 | -0.162 | 0.040 | 4.44e-05 | 4.06e-01 |
| 2:112447888 | rs10174353 | C/T | 0.448 | 0.142 | 0.035 | 4.49e-05 | 4.81e-02 |
| 2:105536116 | rs2889336 | G/C | 0.286 | -0.163 | 0.040 | 4.54e-05 | 2.75e-01 |
| 2:96855241 | rs4907230 | A/G | 0.323 | 0.152 | 0.037 | 4.55e-05 | 4.40e-01 |
| 2:105557895 | rs2033303 | T/C | 0.273 | -0.161 | 0.039 | 4.57e-05 | 3.78e-01 |
| 2:218227288 | rs78096412 | G/T | 0.098 | -0.237 | 0.058 | 4.61e-05 | 1.17e-01 |
| 2:218157502 | rs74899380 | C/G | 0.109 | -0.229 | 0.056 | 4.65e-05 | 4.77e-01 |
| 2:105550991 | rs72832219 | C/A | 0.271 | -0.162 | 0.040 | 4.78e-05 | 3.03e-01 |
| 2:112441433 | rs6728061 | A/G | 0.449 | 0.140 | 0.034 | 4.80e-05 | 1.60e-02 |
| 2:218227410 | rs73991072 | T/C | 0.097 | -0.237 | 0.058 | 4.95e-05 | 1.17e-01 |
| 2:96777340 | rs7604842 | C/T | 0.342 | 0.147 | 0.036 | 5.06e-05 | 3.90e-01 |
| 2:218222372 | rs28542381 | A/G | 0.101 | -0.230 | 0.057 | 5.06e-05 | 1.17e-01 |
| 2:112447296 | rs55937046 | G/A | 0.449 | 0.141 | 0.035 | 5.07e-05 | 4.75e-02 |
| 2:218157508 | rs74631012 | G/A | 0.109 | -0.228 | 0.056 | 5.07e-05 | 4.66e-01 |
| 2:218188700 | rs10804257 | C/T | 0.113 | -0.222 | 0.055 | 5.11e-05 | 4.43e-01 |
| 2:96774786 | rs10183151 | T/G | 0.342 | 0.146 | 0.036 | 5.35e-05 | 4.13e-01 |
| 2:105544888 | rs6734108 | A/G | 0.27 | -0.161 | 0.040 | 5.52e-05 | 3.72e-01 |
| 2:96822373 | rs1030864 | G/A | 0.341 | 0.146 | 0.036 | 5.76e-05 | 3.73e-01 |
| 2:105559947 | rs6705953 | C/T | 0.273 | -0.159 | 0.039 | 5.87e-05 | 4.12e-01 |
| 2:160352115 | rs13003356 | C/T | 0.093 | 0.243 | 0.060 | 5.94e-05 | 2.89e-02 |
| 2:218226516:1 | rs77820558 | C/T | 0.099 | -0.231 | 0.058 | 5.98e-05 | 1.20e-01 |
| 2:218194886 | rs56163890 | A/G | 0.112 | -0.222 | 0.055 | 6.02e-05 | 3.28e-01 |
| 2:96825363 | rs1168968 | A/G | 0.34 | 0.145 | 0.036 | 6.31e-05 | 3.50e-01 |
| 2:112451387 | rs3860380 | G/A | 0.449 | 0.140 | 0.035 | 6.40e-05 | 4.55e-02 |
| 2:96745729 | rs2692936 | A/G | 0.348 | 0.144 | 0.036 | 6.50e-05 | 6.59e-01 |
| 2:96880147 | rs58448550 | T/G | 0.324 | 0.148 | 0.037 | 6.50e-05 | 4.70e-01 |
| 2:96751871 | rs2692937 | A/G | 0.347 | 0.144 | 0.036 | 6.64e-05 | 6.52e-01 |
| 2:96737083 | rs2579552 | G/A | 0.347 | 0.143 | 0.036 | 7.03e-05 | 6.37e-01 |
| 2:96756547 | rs2692893 | T/C | 0.346 | 0.144 | 0.036 | 7.09e-05 | 5.54e-01 |
| 2:112437140 | rs1464095 | G/A | 0.449 | 0.135 | 0.034 | 7.47e-05 | 1.20e-02 |
| 2:96741944 | rs2579550 | A/G | 0.347 | 0.143 | 0.036 | 7.49e-05 | 6.82e-01 |
| 2:96742833 | rs2579549 | G/T | 0.347 | 0.143 | 0.036 | 7.49e-05 | 6.82e-01 |
| 2:105531516 | rs34489771 | T/G | 0.265 | -0.160 | 0.040 | 7.61e-05 | 3.58e-01 |
| 2:218177312 | rs55884900 | G/A | 0.121 | -0.208 | 0.052 | 7.78e-05 | 5.73e-01 |
| 2:218162330 | rs78665806 | G/A | 0.12 | -0.210 | 0.053 | 7.85e-05 | 5.56e-01 |
| 2:112440772 | rs4459742 | G/A | 0.446 | 0.137 | 0.035 | 7.94e-05 | 1.12e-02 |
| 2:96737860 | rs2692934 | T/C | 0.347 | 0.142 | 0.036 | 8.07e-05 | 6.59e-01 |
| 2:218159244 | rs6752033 | T/C | 0.16 | -0.191 | 0.048 | 8.12e-05 | 6.99e-01 |
| 2:96813480 | rs1168976 | A/G | 0.341 | 0.143 | 0.036 | 8.12e-05 | 3.66e-01 |
| 2:96814075 | rs1168975 | A/G | 0.341 | 0.143 | 0.036 | 8.12e-05 | 3.66e-01 |
| 2:96814928 | rs1168974 | A/G | 0.341 | 0.143 | 0.036 | 8.12e-05 | 3.66e-01 |
| 2:96815492 | rs1168972 | A/G | 0.341 | 0.143 | 0.036 | 8.12e-05 | 3.66e-01 |
| 2:96816606 | rs1168970 | T/C | 0.341 | 0.143 | 0.036 | 8.12e-05 | 3.66e-01 |
| 2:179039491 | rs334128 | T/G | 0.422 | 0.141 | 0.036 | 8.25e-05 | 2.66e-02 |
| 2:218181721 | rs79286679 | C/T | 0.116 | -0.213 | 0.054 | 8.35e-05 | 4.05e-01 |
| 2:228136823 | rs12619141 | T/A | 0.148 | -0.190 | 0.048 | 8.58e-05 | 5.21e-02 |
| 2:228137049 | rs12619189 | G/A | 0.148 | -0.190 | 0.048 | 8.58e-05 | 5.21e-02 |
| 2:228137357 | rs78908239 | A/T | 0.147 | -0.189 | 0.048 | 8.82e-05 | 4.39e-02 |
| 2:105563377 | rs113534447 | A/G | 0.271 | -0.155 | 0.040 | 8.91e-05 | 4.38e-01 |
