# Supplementary material for: Bivariate genome-wide association analysis strengthens the role of bitter receptor clusters on chromosomes 7 and 12 in human bitter taste
Source: BMC Genomics. 2018 Sep 17;19:678. doi: 10.1186/s12864-018-5058-2 (PMC6142396; doi:10.1186/s12864-018-5058-2)
Supplement: Supplementary file 8 — Table S8. Mean, standard deviation, and heritability estimates for the perceived intensity of bitter tastes. (DOCX 47 kb) [file 12864_2018_5058_MOESM8_ESM.docx]

**Table S8. Mean, standard deviation, and heritability estimates for the perceived intensity of bitter tastes.**

| Trait | Mean | SD | Heritability | SE |
| --- | --- | --- | --- | --- |
| PROP | 36.54 | 29.73 | 0.71 | 0.03 |
| PROP paper | 38.82 | 28.95 | 0.40 | 0.04 |
| Quinine | 46.03 | 22.77 | 0.38 | 0.05 |
| Caffeine | 52.32 | 23.28 | 0.31 | 0.05 |
| SOA | 52.03 | 22.62 | 0.40 | 0.05 |
| DB | 79.50 | 24.77 | 0.45 | 0.05 |

PROP, propylthiouracil. SOA, sucrose octaacetate. DB, denatonium benzoate. SD/SE, standard deviation/error. Perceived intensity were ratings on general Labelled Magnitude Scale (gLMS). Heritabilities were estimated using GEMMA based on genetic relatedness matrix.
