# Supplementary material for: Bivariate genome-wide association analysis strengthens the role of bitter receptor clusters on chromosomes 7 and 12 in human bitter taste
Source: BMC Genomics. 2018 Sep 17;19:678. doi: 10.1186/s12864-018-5058-2 (PMC6142396; doi:10.1186/s12864-018-5058-2)
Supplement: Supplementary file 9 — Figure S1. Direction and size of the effects of SNP associations on the perceived intensities of quinine, caffeine, sucrose octaacetate (SOA) and denatonium benzoate (DB). (DOCX 240 kb) [file 12864_2018_5058_MOESM9_ESM.docx]

**
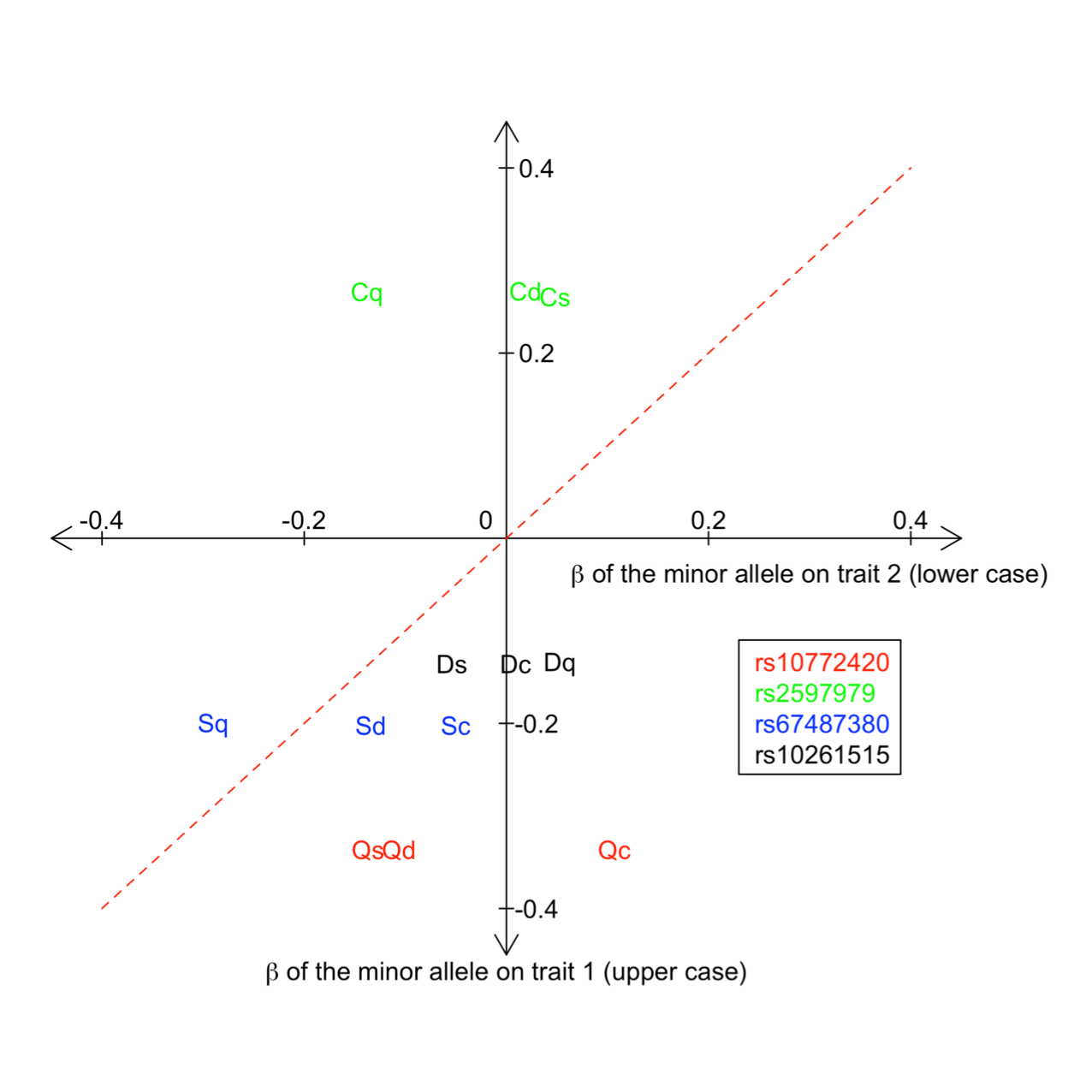
**

**Figure S1. Direction and size of the effects of SNP associations on the perceived intensities of quinine, caffeine, sucrose octaacetate (SOA) and denatonium benzoate (DB).** rs10772420 has the strongest effect on quinine is in the same direction (negative) as its effect on SOA and DB (Qs and Qd) but the opposite (positive) on caffeine (Qc). The effect size (β; see Supplementary Table 9 for variance explained) is the largest for quinine, and it is smaller but at a similar level for the others. The minor allele of rs2597979 has opposite effects on caffeine and quinine (Cq). The direction of effect on SOA and DB (Cs and Cd) is the same as that on caffeine but the size of their effects is subtle. The minor allele of rs67487380 has negative effects on all bitter tastes. The effect size for SOA is similar to those for quinine and DB (Sq and Sd), which positions them close to the diagonal line. The effect on caffeine is the minimum. The minor allele of rs10261515 has the largest and negative effect on DB. Its effects on the others are subtle, but they tend to be negative, null and positive on SOA, caffeine and quinine (Ds, Dc and Dq), respectively. add effect sizes for each SNPs.
