# Supplementary material for: Bivariate genome-wide association analysis strengthens the role of bitter receptor clusters on chromosomes 7 and 12 in human bitter taste
Source: BMC Genomics. 2018 Sep 17;19:678. doi: 10.1186/s12864-018-5058-2 (PMC6142396; doi:10.1186/s12864-018-5058-2)
Supplement: Supplementary file 10 — Table S9. Phenotypic and genetic variance in the perceived intensity of quinine, caffeine, sucrose octaacetate (SOA) and denatonium benzoate (DB) explained by rs10772420, rs2597979, rs67487380 and rs10261515. (DOCX 51 kb) [file 12864_2018_5058_MOESM10_ESM.docx]

**Table S9. Phenotypic and genetic variance in the perceived intensity of quinine, caffeine, sucrose octaacetate (SOA) and denatonium benzoate (DB) explained by rs10772420, rs2597979, rs67487380 and rs10261515.**

|  | Phenotypic variance explained (%) | | | |  | Genetic variance explained (%) | | | | |
| --- | --- | --- | --- | --- | --- | --- | --- | --- | --- | --- |
| SNP | Quinine | Caffeine | SOA | DB |  | Quinine | Caffeine | SOA | DB |  |
| rs10772420 | 5.67 | 0.57 | 0.94 | 0.56 |  | 14.92 | 1.49 | 2.46 | 1.48 |  |
| rs2597979 | 0.52 | 1.91 | 0.05 | 0.02 |  | 1.69 | 6.15 | 0.16 | 0.05 |  |
| rs67487380 | 3.36 | 0.10 | 1.63 | 0.72 |  | 8.40 | 0.25 | 4.07 | 1.79 |  |
| rs10261515 | 0.14 | 0.00 | 0.15 | 0.93 |  | 0.31 | 0.01 | 0.32 | 2.06 |  |
