# Supplementary material for: Bivariate genome-wide association analysis strengthens the role of bitter receptor clusters on chromosomes 7 and 12 in human bitter taste
Source: BMC Genomics. 2018 Sep 17;19:678. doi: 10.1186/s12864-018-5058-2 (PMC6142396; doi:10.1186/s12864-018-5058-2)
Supplement: Supplementary file 11 — Figure S2. Univariate GWAS for the perception of (a) PROP solution (n = 1757) and (b) PROP paper (n = 1999). Left part are Manhattan plots displaying the association P-value for each SNP in the genome (displayed as –log10 of the P-value). The red line indicates the genome-wide significance threshold of P = 5.0e-8. Right part are regional plots ±400kb from the top SNPs on chromosome 7 for PROP solution and chromosome 2 for PROP paper with the gene model below. (DOCX 444 kb) [file 12864_2018_5058_MOESM11_ESM.docx]

**
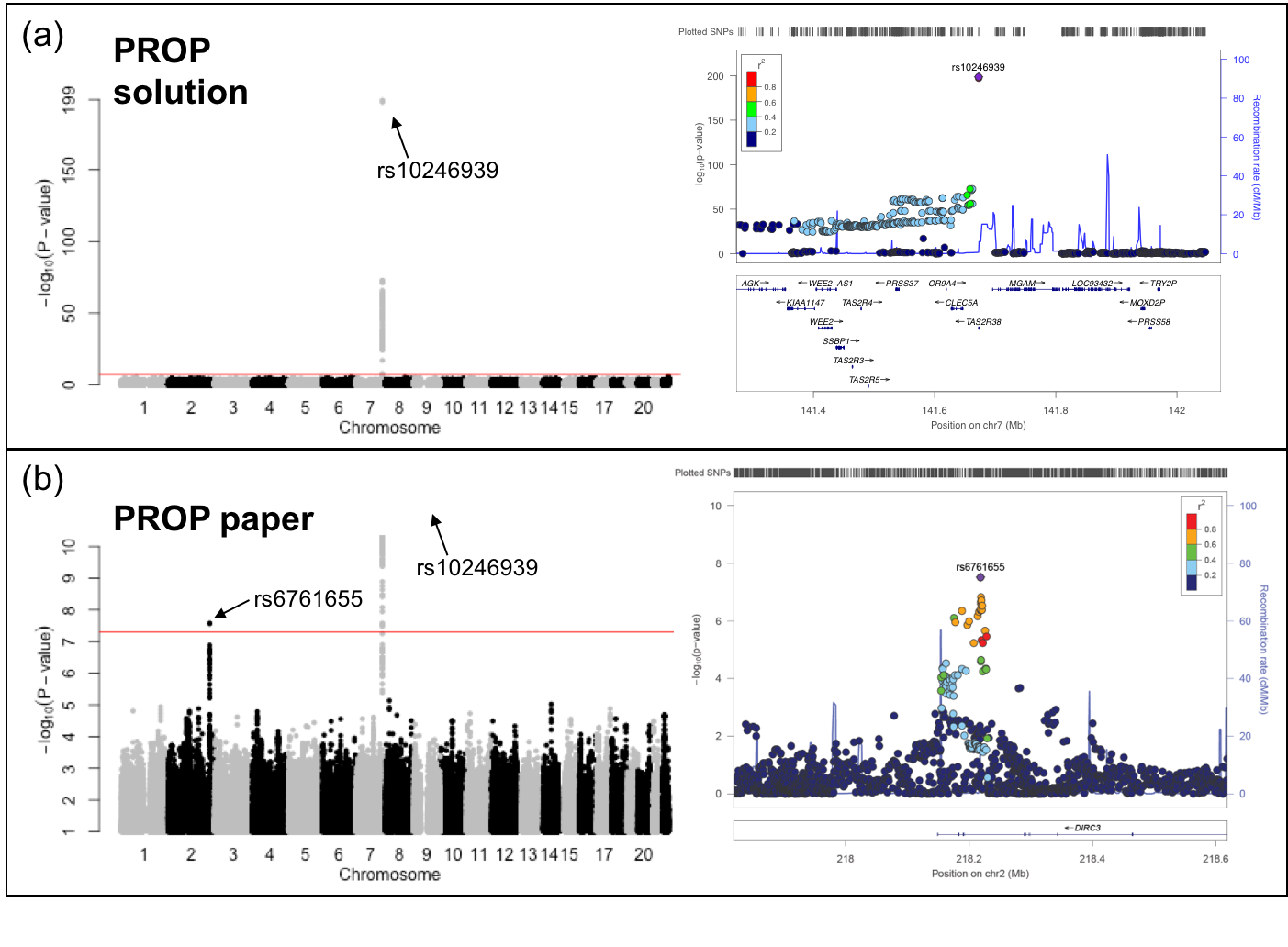
**

**Figure S2. Univariate GWAS for the perception of (a) PROP solution (n = 1757) and (b) PROP paper (n = 1999).** Left part are Manhattan plots displaying the association P-value for each SNP in the genome (displayed as –log_10_ of the P-value). The red line indicates the genome-wide significance threshold of P = 5.0e-8. Right part are regional plots ±400kb from the top SNPs on chromosome 7 for PROP solution and chromosome 2 for PROP paper with the gene model below.
