# Supplementary material for: Bivariate genome-wide association analysis strengthens the role of bitter receptor clusters on chromosomes 7 and 12 in human bitter taste
Source: BMC Genomics. 2018 Sep 17;19:678. doi: 10.1186/s12864-018-5058-2 (PMC6142396; doi:10.1186/s12864-018-5058-2)
Supplement: Supplementary file 12 — Figure S3. Univariate GWAS for the perception of PROP paper from our previous GWAS of 1756 Australian adolescents. A Manhattan plot displays the association P-value for each SNP in the genome (displayed as –log10 of the P-value). This figure is the Fig. 1b from our previous published paper (PMID: 20675712). (DOCX 292 kb) [file 12864_2018_5058_MOESM12_ESM.docx]

**
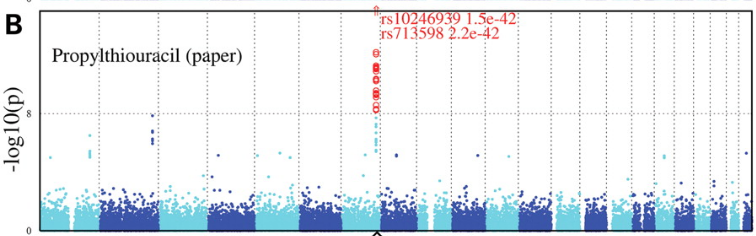
**

**Figure S3. Univariate GWAS for the perception of PROP paper from our previous GWAS of 1756 Australian adolescents.** A Manhattan plot displays the association P-value for each SNP in the genome (displayed as –log_10_ of the P-value). This figure is the Figure 1B from our previous published paper (PMID: 20675712).
