# Supplementary material for: Bivariate genome-wide association analysis strengthens the role of bitter receptor clusters on chromosomes 7 and 12 in human bitter taste
Source: BMC Genomics. 2018 Sep 17;19:678. doi: 10.1186/s12864-018-5058-2 (PMC6142396; doi:10.1186/s12864-018-5058-2)
Supplement: Supplementary file 13 — Table S10. Top PROP paper-associated SNPs on chromosome 2 in the current study (n = 1999) and their associations in GWAS of (1) 225 subjects from the general population of the São Paulo metropolitan area of Brazil, (2) 466 subjects from Silk Road and (3) 2588 subjects from three Italian cohorts. (DOCX 104 kb) [file 12864_2018_5058_MOESM13_ESM.docx]

**Table S10. Top PROP paper-associated SNPs on chromosome 2 in the current study (n = 1999) and their associations in GWAS of (1) 225 subjects from the general population of the São Paulo metropolitan area of Brazil, (2) 466 subjects from Silk Road and (3) 2588 subjects from three Italian cohorts.**

| SNP | Chr:Position | A1/A2 | MAF | β | SE | r^2^ | P_Current Study | P_Brazilian Sample | P_Silk Road | P_Italian Sample |
| --- | --- | --- | --- | --- | --- | --- | --- | --- | --- | --- |
| rs6761655 | 2:218218646 | G/A | 0.186 | -0.246 | 4.41e-2 | 1.83% | 2.69e-8 | NA | 0.95 | 0.99 |
| rs6736242 | 2:218218695 | A/G | 0.186 | -0.246 | 4.41e-2 | 1.83% | 2.69e-8 | NA | - | - |
| rs7586502 | 2:218219311 | A/G | 0.142 | -0.259 | 4.88e-2 | 1.63% | 1.31e-7 | NA | - | - |
| rs80312552 | 2:218220180 | G/A | 0.140 | -0.258 | 4.91e-2 | 1.60% | 1.70e-7 | NA | - | - |
| rs6435978 | 2:218219166 | T/C | 0.142 | -0.256 | 4.89e-2 | 1.59% | 1.85e-7 | 0.87 | - | - |
| rs6435976 | 2:218219074 | T/C | 0.142 | -0.253 | 4.87e-2 | 1.56% | 2.15e-7 | NA | - | - |
| rs6435977 | 2:218219139 | A/G | 0.143 | -0.254 | 4.88e-2 | 1.58% | 2.18e-7 | NA | - | - |
| rs4674157 | 2:218221469 | A/G | 0.140 | -0.255 | 4.93e-2 | 1.56% | 2.59e-7 | NA | - | - |
| rs4674155 | 2:218220849 | A/G | 0.139 | -0.252 | 4.94e-2 | 1.52% | 3.60e-7 | NA | - | - |
| rs4674156 | 2:218220860 | T/C | 0.139 | -0.252 | 4.94e-2 | 1.52% | 3.60e-7 | NA | - | - |
| rs6707253 | 2:218218855 | G/A | 0.142 | -0.248 | 4.87e-2 | 1.50% | 3.77e-7 | NA | - | - |
| rs6707229 | 2:218218774 | C/A | 0.143 | -0.248 | 4.87e-2 | 1.50% | 4.00e-7 | NA | - | - |
| rs13023129 | 2:218188539 | C/T | 0.232 | -0.211 | 4.19e-2 | 1.59% | 4.99e-7 | NA | - | - |
| rs1863193 | 2:218216220 | C/T | 0.211 | -0.215 | 4.27e-2 | 1.54% | 5.25e-7 | NA | - | - |
| rs13008830 | 2:218214057 | C/T | 0.211 | -0.214 | 4.30e-2 | 1.53% | 6.69e-7 | NA | - | - |
| rs4141835 | 2:218175472 | A/G | 0.231 | -0.205 | 4.14e-2 | 1.49% | 8.12e-7 | 0.95 | - | - |

We give allele frequency and effect sizes with reference to allele A1. Base-pair position is based on GRCh37; A1/A2, minor/major allele; MAF, minor allele frequency; β, the effect size; SE, standard error of the β; r^2^, percent variance of the trait accounted for by the SNP; NA, SNP information was not available. The PMID is 22132133 for the GWAS of the Brazilian sample. Data from the Silk Road and Italian samples are unpublished and obtained through personal communication with Dr. Robino Antonietta from the Italian Ministry of Health. The top SNP rs6761655 was in high LD (r^2^ > 0.6) with all other SNPs with P < 1e-6, and is a good indicator of the signal on chromosome 2.
