# Supplementary material for: Bivariate genome-wide association analysis strengthens the role of bitter receptor clusters on chromosomes 7 and 12 in human bitter taste
Source: BMC Genomics. 2018 Sep 17;19:678. doi: 10.1186/s12864-018-5058-2 (PMC6142396; doi:10.1186/s12864-018-5058-2)
Supplement: Supplementary file 14 — Table S11. Results of the annotation conducted with HaploReg v4.1. (DOCX 119 kb) [file 12864_2018_5058_MOESM14_ESM.docx]

| Chr | pos_hg38 | Lead_SNP, Trait^a^ | Non-syn SNPs in LD^b^ | CR^c^ | DNAse^d^ | Histone marks^e^ | Motifs changed^f^ | eQLT^g^ | mQTL^h^ | GENCODE genes |
| --- | --- | --- | --- | --- | --- | --- | --- | --- | --- | --- |
| 7 | 141972804 | rs10246939, PROP solution & paper | Ala49Pro [rs713598] and Ile296Val [rs10246939] in *TAS2R38* | ✓ | Pancreas | Enhancer  In skin and blood | DMRT7, Evi-1_4, KAP1, NF-Y, PLZF, Pou2f2, PU.1, Smad3, Sox | CLEC5A, KIAA1147, TAS2R5, TAS2R38, WEE2-AS1, ENSG00000257093.2_141356528_141362651, ENSG00000257093.2_141363972_141364040, ENSG00000257093.2_141364704_141364854, ENSG00000257093.2_141385278_141385436, ENSG00000228775 |  | MGAM, TAS2R38 |
| 12 | 11020856 | rs10772420, quinine | Arg299Cys [rs10772420] in *TAS2R19* |  | Skin |  | Maf | PRH1, PRR4, TAS2R10, TAS2R14, TAS2R19, TAS2R20, TAS2R31, TAS2R43, TAS2R50, TAS2R64P,  ENSG00000212124.2_11174218_11175219 | chr12:11067603-11067653 [hg18 coord probe cg25677688] TAS2R19 | TAS2R19 |
| 12 | 11037367 | rs2597979, caffeine | Leu162Met, Leu162Val [rs10743938] in *TAS2R31* |  |  |  | NRSF, PU.1, SPIB | PRR4, RP11-785H5.1, TAS2R14, TAS2R15, TAS2R20, TAS2R31, TAS2R43, TAS2R45, TAS2R64P, ENSG00000231887.2_11035134_11035297, | chr12:11030521-11030571 [hg18 coord probe cg11032157] TAS2R19, chr12:11067603-11067653 [hg18 coord probe cg25677688] TAS2R50 | PRR4, TAS2R31 |
| 12 | 11041785 | rs67487380, SOA |  |  |  |  | BDP1, CTCF, GLI, HNF4, Ifr, LXR, NRSF, Rad21, Pax-4, RXRA, RXRA, SMC3, TATA, Znf143 | RP11-434C1.1, RP11-785H5.1, RP11-785H5.2, PRB2, PRR4, TAS2R10, TAS2R12, TAS2R14, TAS2R15, TAS2R19, TAS2R20, TAS2R31, TAS2R43, TAS2R46, TAS2R64P, ENSG00000111215.5_10999644_10999966, ENSG00000212124.2_11174218_11175219, ENSG00000255837.1_11149094_11150474, ENSG00000256436.1_11182986_11184006 |  | PRR4 |
| 7 | 141698907 | rs10261515, DB |  | ✓ | Blood, immune cell, stem cell, brain, heart, stomach, muscle. | Promoter in lung; enhancer in stem cell, immune cell, liver, brain, stomach, blood, colon, heart, lung. | CDP, Hoxa5 | AC004918.1-1\|KIAA1147, AGK, CD1B, CLEC5A, FLJ40852, hmm33189, KIAA1147, LCHN, LOC254719, OR9A3P, PRSS37, RP11-744I24.2, RP5-894A10.6, TAS2R4, TAS2R5, WEE2, WEE2-AS1, ENSG00000127366.4_141490017_141491166, ENSG00000257093.2_141356528_141362651, ENSG00000257093.2_141363972_141364040, ENSG00000257093.2_141364704_141364854, ENSG00000257093.2_141364987_141365118, ENSG00000257093.2_141366087_141366225, ENSG00000257093.2_141373867_141374020, ENSG00000257093.2_141385278_141385436, ENSG00000257093.2_141386359_141386458, ENSG00000257093.2_141401686_141401953, ENSG00000228775, ENSG00000257093.2_141356528_141362651 |  | KIAA1147 |

**Table S11. Results of the annotation conducted with HaploReg v4.1.**

pos_hg38, base-pair position in the Genome Reference Consortium human genome build 38; CR, conserved region; eQTL, expression quantitative trait loci; LD, linkage disequilibrium; mQTL, methylation quantitative trait loci; SNP, single-nucleotide polymorphism. SOA, sucrose octa-acetate; DB, dentonium benzoate. ^a^Lead SNP and the associated bitter taste. ^b^Non-synonymous SNPs in LD (CEU: r^2^⩾0.80) with lead SNP. ^c^Check marks (✓) denote the presence of a conserved region (spanning lead SNP and its correlated proxies, CEU: r^2^⩾ 0.8). ^d^Tissues with the presence of DNase-I hypersensitivity sites at region spanning lead SNP and its correlated proxies, CEU: r^2^⩾ 0.8. DNAse hypersensitivity sites are related to transcriptional activity because these are chromatin regions that are less condensed and more accessible to transcription factors. ^f^Enhancer (H3K4me1) or promoter (H3K4me3) histone marks spanning lead SNP and its correlated proxies, CEU: r^2^⩾ 0.8. Enhancer: a short region of DNA (50-1500 bp) that can be bound by proteins (activators) to increase the transcription of a gene. An enhancer can be 1MB upstream/downstream of its targeted gene. Promoter: a region of DNA (100-1000 bp) that initiates transcription of a gene. A promoter is located near transcription start site (5’) of a gene. ^f^Regulatory motifs altered by lead SNP and its correlated proxies. Regulatory motifs are short nucleotide sequences typically upstream of genes where transcription factors bind to control the expression of genes. ^g^Expression QTL (*cis*-eQTL, variants are within 1 Mb up- and downstream of the transcription start site) for lead SNP and its correlated proxies, CEU: r^2^⩾ 0.8, derived from eQTL studies including results with P < 1e-5 from the GTEx (Genotype-Tissue Expression) and GRASP (Genome-Wide Repository of Associations Between SNPs and Phenotypes). Tissues include esophagus, muscle, salivary gland, lung, heart, ovary, uterus, artery, fallopian tube, vagina, skin, cervix, bladder, thyroid, adipose, colon, breast, testis, nerve, small intestine, pituitary, pancreas, colon, stomach, prostate, adrenal gland, liver, spleen, and kidney. ^h^Methylation QTLs for lead SNP derived from temporal cortex, frontal cortex and caudal pons. The results are obtained using GRASP. All associations have P < 1e-8.

Brief summary

1. PROP, quinine, caffeine, and SOA-associated SNPs include missense variants.
2. PROP, quinine, and DB-associated SNPs are located within conserved region.
3. Except for caffeine-associated SNPs, all SNPs are in high DNAse hypersensitivity sites.
4. PROP, SOA, and DB-associated SNPs are inside promoter and/or enhancer regions.
5. All SNPs may cause regulatory motif change of a protein.
6. All SNPs are eQTLs.
7. Quinine, caffeine, and SOA-associated SNPs are mQTLs.
8. None of these analyses was performed using taste tissues.
