# Supplementary material for: Bivariate genome-wide association analysis strengthens the role of bitter receptor clusters on chromosomes 7 and 12 in human bitter taste
Source: BMC Genomics. 2018 Sep 17;19:678. doi: 10.1186/s12864-018-5058-2 (PMC6142396; doi:10.1186/s12864-018-5058-2)
Supplement: Supplementary file 15 — Table S12. Genetic variants associated with human bitter taste perception using the sample with one member of each MZ pair removed (see Table 1 for associations using the full sample). (DOCX 24 kb) [file 12864_2018_5058_MOESM15_ESM.docx]

**Table S12. Genetic variants associated with human bitter taste perception using the sample with one member of each MZ pair removed (see Table 1 for associations using the full sample).**

| **Trait 1** | **SNP** | **Chr:Position** | **A1/A2** | **MAF** | **β** | **SE** | **r^2^** | **P** | **Trait 2** | | | |
| --- | --- | --- | --- | --- | --- | --- | --- | --- | --- | --- | --- | --- |
|  |  |  |  |  |  |  |  |  | **Quinine** | **Caffeine** | **SOA** | **DB** |
|  |  |  |  |  |  |  |  |  | **P_bivariate** | | | |
| Quinine | rs10772420 | 12:11174276 | G/A | 0.470 | -0.346 | 0.035 | 5.96% | **2.8e-22*** | - | **6.7e-60*** | **3.7e-24*** | **1.6e-24*** |
| Caffeine | rs2597979**^†^** | 12:11189966 | G/C | 0.165 | 0.277 | 0.050 | 2.11% | **3.0e-8** | **1.2e-22*** | - | **1.2e-10*** | **2.8e-11*** |
| SOA | rs67487380 | 12:11194384 | A/G | 0.275 | -0.205 | 0.041 | 1.68% | 6.0e-7 | **1.2e-13*** | 2.6e-7 | - | 3.1e-6 |
| DB | rs10261515 | 7:141398707 | G/A | 0.493 | -0.140 | 0.038 | 0.98% | 2.9e-4 | 9.0e-7 | 7.2e-6 | 7.8e-4 | - |
| PROP solution | rs10246939 | 7:141672604 | C/T | 0.436 | 0.974 | 0.029 | 46.69% | **1.1e-188*** |  |  |  |  |
| PROP paper | rs10246939 | 7:141672604 | C/T | 0.436 | 0.545 | 0.033 | 14.62%^a^ | **3.7e-56*** |  |  |  |  |
| PROP paper | rs6761655**^‡^** | 2:218218646 | G/A | 0.186 | -0.252 | 0.045 | 1.92% | **4.2e-8** |  |  |  |  |
| We report the associations for same SNPs from Table 1. SNPs that were not identified in our previous GWAS are underlined. Allele frequency and effect sizes are reported with reference to allele A1. Base-pair position is based on GRCh37; A1/A2, minor/major allele; MAF, minor allele frequency; β, the effect size; SE, standard error of the β; r^2^, percent variance of the trait accounted for by the SNP; P, P-value from the univariate association analysis of trait 1; P_bivariate, P-value from the bivariate association analysis of traits 1 and 2; SOA, sucrose octaacetate; DB, denatonium benzoate; **bold**, P < genome-wide significance threshold of 5.0e-8; *, P < corrected significance threshold of 1.0e-8; **^†^**, an independent replication; **^‡^**, no evidence of replication. | | | | | | | | | | | | |
