# Supplementary figures and images for: Bivariate genome-wide association analysis strengthens the role of bitter receptor clusters on chromosomes 7 and 12 in human bitter taste
Source: BMC Genomics. 2018 Sep 17;19:678. doi: 10.1186/s12864-018-5058-2 (PMC6142396; doi:10.1186/s12864-018-5058-2)

**
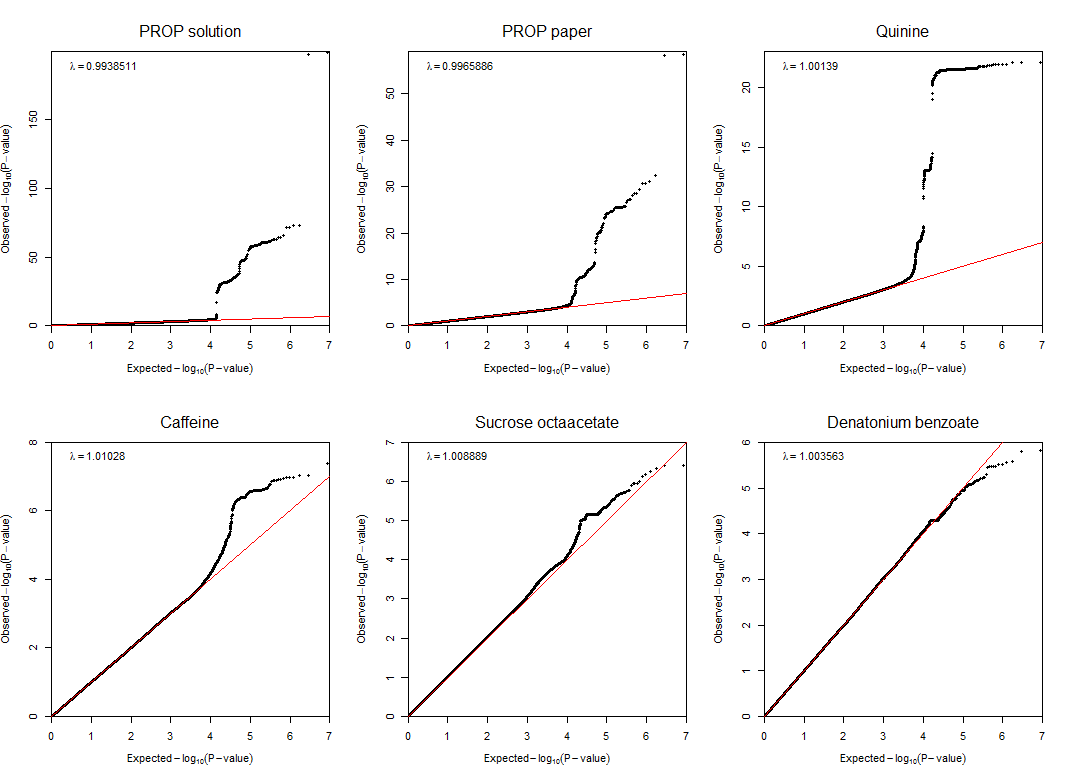
**

**Figure S4. The Q-Q plots for each of the univariate analyses.** PROP: propylthiouracil.

Supplement: Supplementary file 16 — Figure S4. The Q-Q plots for each of the univariate analyses. PROP: propylthiouracil. (DOCX 2545 kb) [file 12864_2018_5058_MOESM16_ESM.docx]
