# Supplementary material for: Bivariate genome-wide association analysis strengthens the role of bitter receptor clusters on chromosomes 7 and 12 in human bitter taste
Source: BMC Genomics. 2018 Sep 17;19:678. doi: 10.1186/s12864-018-5058-2 (PMC6142396; doi:10.1186/s12864-018-5058-2)
Supplement: Supplementary file 17 — Figure S5. The Q-Q plots for each of the bivariate analyses. SOA: sucrose octaacetate. DB: denatonium benzoate. (DOCX 2547 kb) [file 12864_2018_5058_MOESM17_ESM.docx]

**
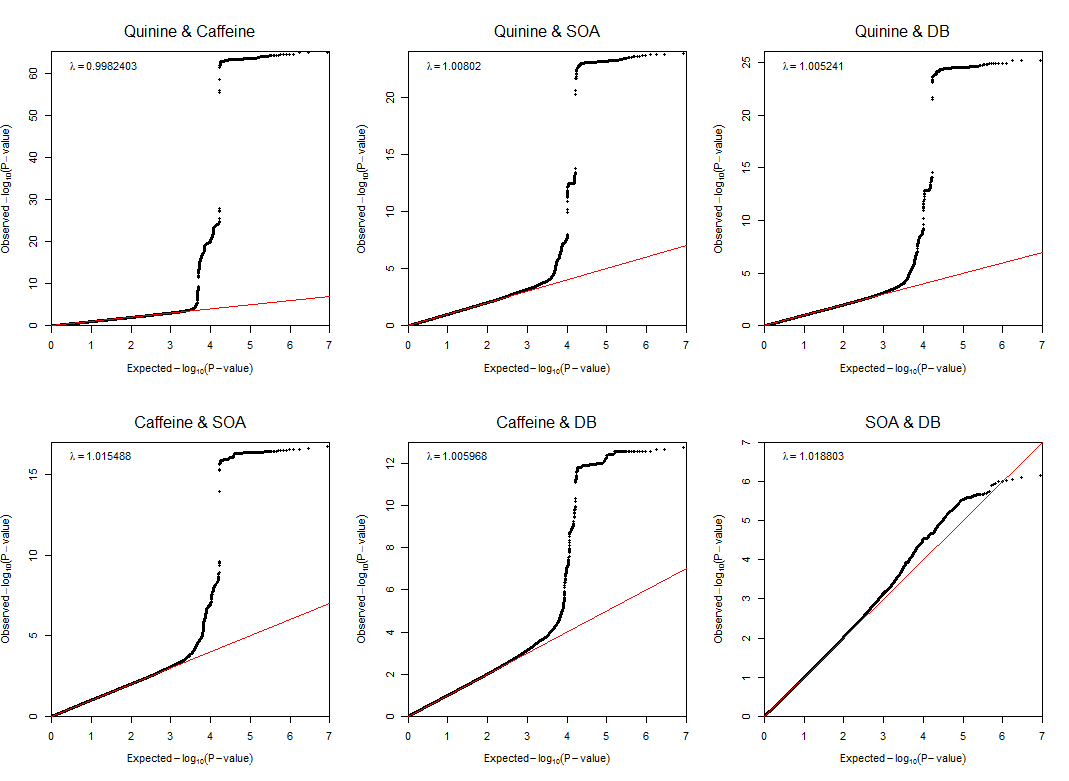
**

**Figure S5. The Q-Q plots for each of the bivariate analyses.** SOA: sucrose octaacetate. DB: denatonium benzoate.
